# MerTK Is Regulated by Orphan Nuclear Receptor 4A1 (NR4A1) and NR4A2 in Colon Cancer Cells

**DOI:** 10.3390/cancers18121993

**Published:** 2026-06-18

**Authors:** Gargi Sivaram, Srijana Upadhyay, Sarah Kakwan, Ahmed Uosef, Maen Abdelrahim, Arafat Rahman Oany, Evan Farkas, Stephen Safe

**Affiliations:** 1 Department of Biochemistry and Biophysics, Texas A&M University, College Station, TX 77843, USA; gs715@tamu.edu; 2 Department of Veterinary Physiology and Pharmacology, Texas A&M University, College Station, TX 77843, USA; supadhyay@cvm.tamu.edu (S.U.); arafatr@tamu.edu (A.R.O.); farkasea@tamu.edu (E.F.); 3 Department of Biomedical Sciences, Texas A&M University, College Station, TX 77843, USA; kakwansarah@tamu.edu; 4 Transplant Immunology, The Houston Methodist Research Institute, Houston, TX 77030, USA; auosef@houstonmethodist.org; 5 Department of Medical Oncology, Houston Methodist Neal Cancer Center, Houston, TX 77030, USA; mabdelrahim@houstonmethodist.org

**Keywords:** MerTK, NR4A1, NR4A2, DIM-3,5 ligands, transcriptional regulation, colon cancer

## Abstract

**Background/Objectives:** The orphan nuclear receptors 4A1 (NR4A1) and NR4A2 are overexpressed in multiple solid tumors, and both receptors exhibit tumor promoter-like activities. A recent study reported that luteolin, a flavonoid that binds NR4A1, decreased the expression of the pro-oncogenic receptor tyrosine kinase MerTK in colon cancer cells. **Methods/Results:** In this study, we observed that MerTK protein was expressed in human SW480 and HCT116 and mouse CT26 colon cancer cell lines, and was significantly downregulated after treatment with 1,1-bis(3′-indolyl)-1-(3,5-disubstitutedphenyl)methane (DIM-3,5) compounds, which are dual NR4A1/NR4A2 ligands. Moreover, knockdown of NR4A1 and NR4A2 also decreased MerTK protein expression and DIM-3,5 ligands, and receptor knockdown also decreased MerTK RNA levels expression. MerTK expression was also downregulated by knockdown of Sp1, Sp3, or Sp4 and by treatment with mithramycin. Subsequent studies using chromatin immunoprecipitation and transfection of a *MERTK* (promoter)–luciferase construct containing transcriptionally active GC-rich promoter elements indicated that MerTK expression in colon cancer cells was regulated by NR4A/Sp complexes, including NR4A1, NR4A2, Sp1, Sp3, and Sp4 transcription factors. **Conclusions:** The participation of NR4A1 and NR4A2 in the regulation of MerTK indicates that DIM-3,5 ligands represent a novel class of agents that can be used to inhibit MerTK expression in cancer cells by acting as dual NR4A1 and NR4A2 inverse agonists.

## Simple Summary

NR4A/Sp transcription factor complexes mediate transactivation of MerTK in colon cancer cells, and this is targeted by DIM-3,5 ligands, which serve as dual NR4A1 and NR4A2 inverse agonists. The TAM kinase MerTK is overexpressed in colon tumors and is a potential drug target. This study demonstrates that the expression of MerTK is coregulated by the orphan nuclear receptors 4A1 (NR4A1) and NR4A2, and that dual NR4A1/NR4A2 DIM-3,5 ligands act as inverse agonists to decrease MerTK mRNA and protein expression. The mechanism of this response was determined by investigating the effects of DIM-3,5 ligands on MerTK promoter–luciferase constructs and chromatin immunoprecipitation assays, and involved both NR4A1 and NR4A2 acting as cofactors of Sp-mediated transactivation. This study demonstrates that DIM-3,5 ligands can act as inhibitors of MerTK by blocking the NR4A1-dependent transcription of this gene.

## 1. Introduction

The TAM receptor tyrosine kinase (RTK) subfamily includes Tyro3, AXL, and Mer (also known as MerTK), and these kinases have multiple functions, which include phagocytic clearance of apoptotic cells, immune cell responses, viral entry, vascular, and pro-carcinogenic activities [1,2,3,4,5,6,7,8]. This subfamily of receptors is widely expressed in normal organs/tissues and is also overexpressed in multiple diseases, including several cancers [5,8,9,10,11]. TAM receptor signaling is primarily dependent on two ligands, namely, growth arrest-specific protein 6 (GAS6) and vitamin K-dependent protein S1 (PROS1) [5,7,12,13]. GAS6 predominantly activates AXL signaling, whereas PROS1 activates Tyro3 and MerTK but not AXL. MerTK and other TAM RTKs are involved in multiple pathways associated with maintaining cellular homeostasis and pathophysiology, including cancer. Results of MerTK-/- (knockout) mouse studies demonstrate a role for this RTK as an anti-inflammatory factor and that this subfamily of receptors modulates immune cell-mediated responses. All three TAM receptors are important for the phagocytic-dependent removal of apoptotic cells, and loss of this function leads to multiple adverse responses, including hyperactive inflammation, retinal damage, and even male infertility. Endothelial cells and platelets required for vascular integrity are also dependent on TAM receptors, and deficiencies in expression of these receptors result in blood vessel dysfunction [14,15,16,17,18]. Overexpression of TAM receptors and their ligands is frequently observed in blood-derived and solid tumors, and AXL is overexpressed in most tumors and activates pathways and genes associated with enhanced tumor growth, survival, metastasis, and immune suppression [5,7,12,13]. Similar results are also observed for MerTK and Tyro3; however, their expression in various tumor types is more limited. There are several ongoing studies on the development and clinical applications of TAM receptor inhibitors, and these are primarily focused on blocking ligand–receptor interactions with antibodies/decoys, inhibiting kinase activities, or inducing TAM degradation using small molecules [19,20,21,22,23,24]. However, targeting MerTK through the inhibition of the transcription of this gene has not been previously explored.

Studies in this laboratory have focused on developing bis-indole-derived compounds (CDIMs) that bind orphan nuclear receptor 4A1 (NR4A1, Nur77) and NR4A2 (Nurr1) [25,26]. In solid tumor-derived cells, CDIMs act as inverse receptor agonists that inhibit NR4A1- and NR4A2-dependent regulation of pro-oncogenic genes that are involved in cell proliferation, survival, migration/invasion, and metastasis [27]. A major NR4A regulatory pathway involves the formation of NR4A/Sp complexes, where the receptor acts as a ligand-dependent cofactor that modulates expression of Sp1- and Sp4-regulated genes [28,29,30,31]. This pathway is not unique to NR4A1 and is observed for most of the 48 nuclear receptors. For example, PD-L1 expression is regulated in breast and colon cancer cells by NR4A1/Sp interactions with a proximal GC-rich sequence in the PD-L1 gene promoter [31]. Previous studies have shown that Sp1 plays a major role in the regulation of MerTK [32], and it has also been reported that the flavonoid luteolin decreases MerTK expression in non-small cell lung cancer cells [33]. Studies in this laboratory reported that luteolin binds NR4A1 [34], suggesting that both Sp1 and possibly NR4A1 may play a role in the expression of MerTK in some cancer cell lines. Results of this study demonstrate, for the first time, that MerTK is regulated by an NR4A/Sp complex in colon cancer cells, and treatment with 1,1-bis(3′-indolyl)-1-(3,5-disubstitutedphenyl)methane (DIM-3,5) analogs that are dual NR4A1/2 ligands downregulates MerTK expression. Thus, MerTK expression is coregulated by NR4A1 and NR4A2, and DIM-3,5 ligands are NR4A1 and NR4A2 inverse agonists that decrease MerTK expression, representing a novel approach for inhibiting this pro-oncogenic tyrosine kinase.

## 2. Materials and Methods

### 2.1. Cell Cultures

SW480 (RRID: CVCL_0546) and HCT-116 (RRID: CVCL_0291) colon cancer cell lines of Homo sapiens origin, and CT26 (RRID: CVCL_7256) colon cancer cell lines of Mus musculus origin were obtained from American Type Culture Collection (Manassas, VA, USA). Cell lines were validated by biosynthesis (Lewisville, TX, USA). SW480 and HCT116 cell lines were maintained in DMEM medium supplemented with 10% FBS (Sigma–Aldrich, Burlington, MA, USA) at 5% CO_2_. The CT26 cell line was maintained in RPMI medium supplemented with 10% FBS (Sigma–Aldrich) at 5% CO_2_.

### 2.2. Reagents

Dual NR4A1 and NR4A2 targeting inverse agonists (DIM-3,5 ligands) 1-bis(3′-indolyl)-1-(3,5-dichlorophenyl)methane (DIM-3,5-CI_2_) and DIM-3-Cl-5-CF_3_ were synthesized by coupling indole and 3,5-dichlorobenzaldehyde as described prievously [25]. The specificity protein (Sp) targeting inhibitor, mithramycin A (Cat: HY-A0122), was purchased from MedChemExpress (Monmouth Junction, NJ, USA). The siRNAs used were purchased from Thermo Fisher (Waltham, MA, USA). Antisense oligos and primers were synthesized and purchased from Integrated DNA Technologies (Coralville, IA, USA). Antibodies used for Western blotting and ChIP assays are detailed in Supplemental Tables S1 and S2. Primers used for qPCR and ChIP assays are detailed in Supplemental Tables S3 and S4. Antibodies used for immunohistochemistry are detailed in Supplemental Table S5.

### 2.3. Western Blotting

Western blotting was used to assess the impact of DIM-3,5 ligands, mithramycin, and NR4A1/2 and Sp1/3/4 knockdowns on protein expression after treatment. Cells were seeded in 12-well plates, in triplicate, at a density of 1.5 × 10^5^ cells per well for drug treatment and allowed to attach overnight. DMSO (control) or DIM-3,5 ligands were added to media supplemented with charcoal-stripped FBS (2.5%) at the indicated concentrations, and cell lysates were extracted after 18 h of treatment. For siRNA and antisense oligo treatment, cells were seeded in 12-well plates, in triplicate, at a density of 7.5 × 10^4^ cells per well and allowed to attach overnight. Transfection of siRNA and antisense oligos (100 nM final concentration) using the RNAiMax transfection reagent (Cat: 13778075, Invitrogen, Waltham, MA, USA) was conducted for 72 h following the manufacturer’s protocol, after which cell lysates were extracted.

Cell lysates were extracted using the high-salt lysis buffer RIPA (Thermo Scientific, Waltham, MA, USA) supplemented with protease and phosphatase inhibitors (GenDEPOT, Baker, TX, USA). Total lysate protein concentration was determined using the Bradford assay protocol on a Beckman DU 640 Spectrophotometer. Western blotting instruments were obtained from BioRad (Hercules, CA, USA). A total of 30 μg of protein from each lysate was loaded onto SDS-polyacrylamide gels. Eight percent gels were used to assess MerTK, NR4A1, NR4A2, mTOR, p-mTOR, and β-Actin expression levels. Ten percent gels were used to assess Sp1, Sp3, Sp4, Bcl-2, and β-Actin expression levels. Gels were transferred onto PVDF membrane at 100 V for 75 min and blocked in 5% milk in TBST for 1 h. Blots were incubated overnight with primary antibodies at 4 °C and washed with Tris-buffered saline and polysorbate 20 (TBST), followed by a 1 h incubation with HRP-conjugated appropriate secondary antibodies at room temperature. Blots were imaged using the Immobilon Western chemiluminescence HRP-substrate (Cat: WBKLS0050, Millipore Sigma, Bedford, MA, USA) on a BioRad Chemidoc MP system (Cat: 1708280, Hercules, CA, USA). Each cell lysate was produced from three biological replicates. The data presented were generated from three independent cell lysate preparations (biological replicates). Data were analyzed using Microsoft Excel and GraphPad Prism version 11 and presented as mean ± SD of three independent cell lysate preparations.

### 2.4. RT-qPCR

Cells were seeded into 6-well plates at a density of 3 × 10^5^ cells per well. Drug treatment was carried out for the indicated time, and knockdown targeting NR4A1/2 was carried out as per the protocol detailed above. mRNA was extracted using the RNeasy Mini Kit (Cat: 74104, Qiagen, Germantown, MD, USA). DNA digestion and mRNA purification were performed using the TURBO DNA-*free* Kit (Cat: 3040986, Invitrogen, Waltham, MA, USA). cDNA was synthesized from purified mRNA using the High-Capacity cDNA Reverse Transcription Kit (Cat: 4368814, Thermo Fisher, Waltham, MA, USA). qPCR was performed using synthesized cDNA with amfiSure qGreen Q-PCR Master Mix and primers listed in Supplemental Table S3 (Cat: Q5601-010, GenDEPOT, Baker, TX, USA) according to the manufacturer’s protocol. Two biological replicates were used to generate each set of purified mRNA and synthesized cDNA. qPCR was performed with three technical replicates and is presented as the relative fold change of these triplicate values using the 2^-ΔΔ-CT^ method [35].

### 2.5. Luciferase Assay

HCT116 and CT26 cells were seeded in 24-well plates at 5 × 10^4^ cells per well. The −388 to 0 region of the *MERTK* promoter was cloned into a mammalian promoter-testing vector (pRP[Pro]-{MerTK}-Luciferase). Purified plasmid was transfected using GeneJuice Transfection Reagent (Cat: 70967, Millipore Sigma, Darmstadt, Germany) and incubated for 24 h. Cells were then treated with DIM-3,5 ligands or mithramycin for 18 h with DMSO as a control. Cell lysates were extracted using Cell Culture Lysis 5X Buffer (Cat: E153A, Promega, Madison, WI, USA), and protein concentrations were determined using Bradford Reagent. Equal amounts of protein were used to assess activity using Luciferase Activity Reagent (E1483, Promega, Madison, WI, USA) and imaged on a BioTek Citation 5 Imaging Reader (Agilent, Santa Clara, CA, USA). Data were generated from three biological replicates. Relative luminescence compared with the DMSO control was measured and presented as the mean ± SD.

### 2.6. ChIP Assay

The chromatin immunoprecipitation (ChIP) assay in the CT26 and HCT116 cell lines was performed using the ChIP-IT Express Magnetic Chromatin Immunoprecipitation Kit (Active Motif, Carlsbad, CA, USA) according to the manufacturer’s protocol. Cells were seeded at 2.5 × 10^7^ density on 15 cm plates and treated with vehicle control (DMSO) or DIM ligands (15 μM) for 4 h. Cells were fixed with 1% formaldehyde, and fixation was stopped with a 0.125 M glycine stop solution. Scraped and collected cells were lysed to collect nuclei. Nuclei were sonicated to the desired chromatin length (100–1500 bp), and chromatin was immunoprecipitated using the antibodies summarized in Supplemental Table S2 and protein G-conjugated magnetic beads overnight at 4 °C. Reverse-crosslinked DNA was purified using Proteinase K digestion. Purefied DNA was assessed using qPCR performed with amfiSure qGreen Q-PCR Master Mix (Cat: Q5601-010, GenDEPOT, Baker, TX, USA) according to the manufacturer’s protocol. The primers are listed in Supplemental Table S4. Sheared chromatin samples for each treatment group were generated from three biological replicates. Data were generated from three technical replicates of these samples and are presented as the relative fold change of these triplicate values using the 2^-ΔΔ-CT^ method [35].

### 2.7. Immunohistochemistry

Colon cancer tissue specimens and matched normal adjacent tissues (NAT) were obtained from five patients (n = 5). Tumor and NAT samples were collected from the same patient to allow paired analysis. All tissues were fixed in formalin, paraffin embedded, and sectioned at 5 μM thickness. Sections were baked at 60 °C for 1 h, deparaffinized in three changes of xylene, and rehydrated through graded ethanol (100%, 90%, 80%, and 70%) to distilled water. Antigen retrieval was performed by heating the sections in 0.01 M sodium citrate buffer (pH 6.0) at 100 °C for 15 min. Endogenous peroxidase activity was blocked using 3% hydrogen peroxide for 10 min at room temperature. Sections were then blocked with 2.5% normal horse serum for 30 min at room temperature. The sections were incubated overnight at 4 °C with primary antibodies against NR4A1, NR4A2, and MerTK (details provided in Supplemental Table S5). Slides were subsequently incubated with ImmPRESS^®^ HRP Horse Anti-Rabbit IgG (Cat: MP-7401, Vector Laboratories, Newark, CA, USA) or Anti-Mouse IgG (Cat: MP-7402, Vector Laboratories, Newark, CA, USA) for 30 min at room temperature. Staining was visualized using DAB (Cat: K3468, Agilent, Santa Clara, CA, USA), and slides were counterstained with hematoxylin, washed with distilled water, dehydrated through graded ethanol (95% and 100%), cleared in xylene, and mounted using Cytoseal 60 (Cat: 831016, Fisher Scientific, Hampton, NH, USA). For each sample, five random, non-overlapping microscopic fields were captured at 20× magnification using a Nikon light microscope. Positive staining was identified by a brown DAB signal. Quantification was performed using ImageJ software (Version 1.53c) with the Color Deconvolution plugin. The percentage of DAB-positive area (% area) relative to the total tissue area was calculated for each field. The mean value of the five fields was used to represent a single sample. Each sample corresponds to one biological replicate (one patient). Data are presented as mean ± SD of five independent patients (n = 5).

### 2.8. Kaplan–Meier Survival Plot Analysis

The Kaplan–Meier Plotter (https://www.kmplot.com/analysis/, accessed on 17 July 2025) is a publicly available online platform for analyzing and evaluating the effects of genes on patient survival outcomes across several cancers [36]. This tool accesses available datasets and is operated by a PostgreSQL server to analyze gene expression and clinical data. This tool was used in this study to analyze the correlation between NR4A1 and NR4A2 gene expression and overall survival (OS) and recurrence-free survival (RFS) in patients with colon cancer. NR4A1 expression was analyzed using the probe 202340_x_at, and NR4A2 was analyzed using the probe 204621_s_at (n = 1061). Kaplan-Meier Plots were generated using mean values for OS and RFS for NR4A1 (520, 538) and NR4A2 (262, 259), respectively, with low- and high-expression groups generated based on median expression.

### 2.9. Statistical Analysis

Preliminary statistical analysis for all data was performed using Microsoft Excel (Microsoft, Redmond, WA, USA). Analysis of variance was performed using GraphPad Prism version 11 (San Diego, CA, USA) by a one-way ANOVA followed by Dunnett’s test for post hoc analysis. GraphPad Prism was also used to generate the graphs in each figure, presented as means ± SD, with individual values and ratios presented where appropriate. The significance levels are defined as follows: (*) for *p* < 0.05, (**) for *p* < 0.01, (***) for *p* < 0.001, (****) for *p* < 0.0001, and (ns) for *p* > 0.05.

## 3. Results

**Figure 1:**
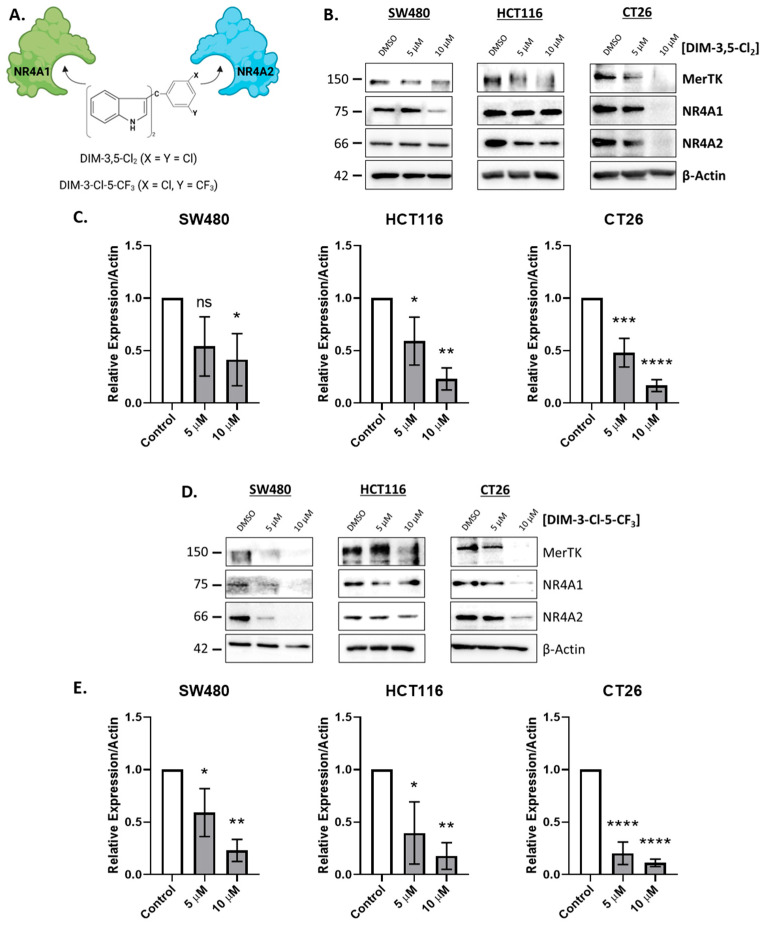
**Treatment of colon cancer cell lines with DIM-3,5 analogs decreases MerTK protein expression.** (**A**) Structure of DIM-3,5 disubstituted dual NR4A1/2 inverse agonists that bind to the ligand-binding domains of NR4A1 and NR4A2. Human and mouse colorectal cancer cell lines were treated with increasing concentrations of (**B**,**C**) DIM-3,5-Cl_2_ and (**D**,**E**) DIM-3-Cl-5-CF_3_ with DMSO as a control for 18 h, and protein expression of NR4A1, NR4A2, and MerTK was determined by Western blotting of whole-cell lysates. (**C**,**E**) Protein expression of MerTK relative to β-actin was quantified for all three cell lines, and results are presented as mean ± SD of three biological replicates as described in Section 2.3. The significance levels are defined as follows: (*) for *p* < 0.05, (**) for *p* < 0.01, (***) for *p* < 0.001, (****) for *p* < 0.0001, and (ns) for *p* > 0.05.

In this study, we investigated the effects of DIM-3,5 bis-indole analogs substituted with 3,5-dichlorophenyl (DIM-3,5-CI_2_) and 3-chloro-5-trifluoromethylphenyl (DIM-3-CI-5-CF_3_) groups on the expression of MerTK in three colon cancer cell lines. These compounds bind to both NR4A1 and NR4A2 and have previously been characterized as dual receptor ligands (Figure 1A) [25]. Treatment with DIM-3,5-CI_2_ decreased MerTK protein levels in human SW480 and HCT116, and mouse CT26 colon cancer cell lines (Figure 1B) by 59%, 77%, and 83%, respectively, at the higher dose level (Figure 1C). Treatment with DIM-3-CI-5-CF_3_ also decreased MerTK expression in all three colon cancer cell lines (Figure 1D) by 80%, 82%, and 89%, respectively (Figure 1E). Both DIM-3,5-Cl_2_ and DIM-3-Cl-5-CF_3_ decreased NR4A1 and NR4A2 protein expression across all cell lines, and DIM-3-Cl-5-CF_3_ was the more effective ligand for inducing receptor degradation in colon cancer cells. The effects on receptor degradation were both ligand- and cell context-dependent, and DIM-3-Cl-5-CF_3_-induced degradation of NR4A1 and NR4A2 in SW480 cells was comparable to that observed using a PROTAC approach in melanoma cells [37].

**Figure 2:**
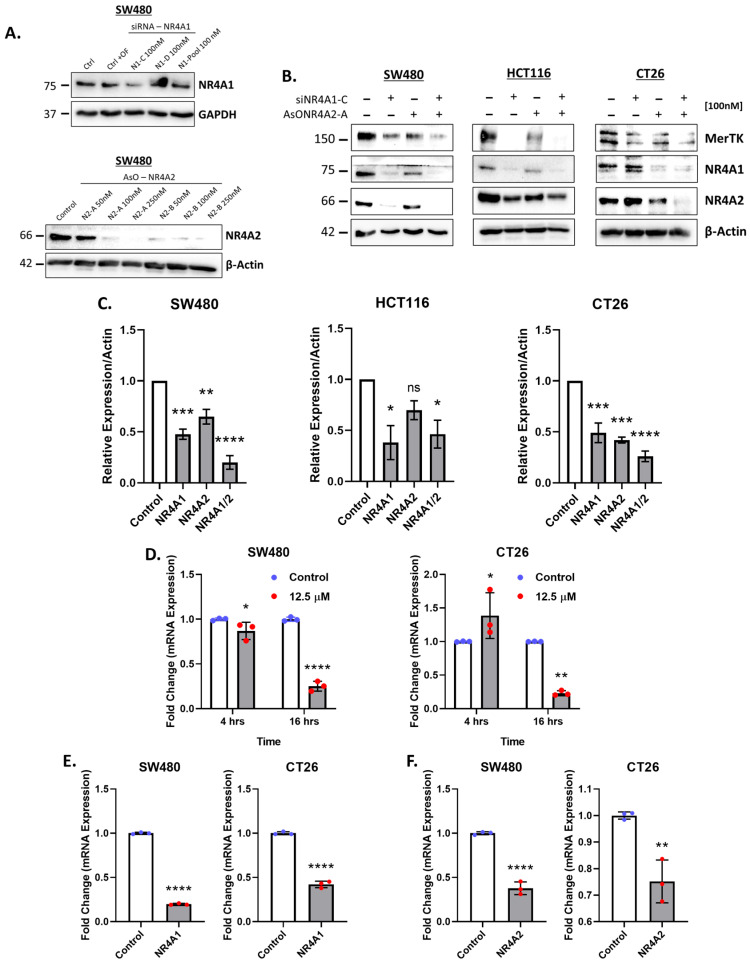
**Effects of receptor knockdown and DIM-3,5 analogs on expression of MerTK in colon cancer cell lines.** Representative human colorectal cancer cells (SW480) were transfected with multiple oligonucleotides (siRNAs) targeting NR4A1 and antisense constructs targeting NR4A2 (**A**), respectively, and whole-cell lysates were analyzed by Western blots. (**B**) Human and mouse colon cancer cell lines were transfected with an siRNA targeting NR4A1 and an antisense oligonucleotide targeting NR4A2, and NR4A1, NR4A2, and MerTK protein expression levels were determined after 72 h using Western blotting of whole-cell lysates. (**C**) Protein expression of NR4A1, NR4A2, and MerTK relative to β-actin was quantified for all three cell lines, and results are presented as mean ± SD of three biological replicates and analyzed as discussed in Section 2.3 and Section 2.8. (**D**) Representative human (SW480) and mouse (CT26) colorectal cancer lines were treated with DIM-3,5-Cl_2,_ and MerTK mRNA levels were determined using qPCR. MerTK mRNA expression after NR4A1 (**E**) and NR4A2 knockdown (**F**) was detected in SW480 and CT26 colon cancer cell lines. Data were quantified using the 2^-ΔΔ-CT^ method as described in Section 2.4 and presented as a means ± SD of three biological replicates. The significance levels are defined as follows: (*) for *p* < 0.05, (**) for *p* < 0.01, (***) for *p* < 0.001, (****) for *p* < 0.0001, and (ns) for *p* > 0.05. Data is graphically represented as follows: Control—blue dots for individual data values and white bars for the mean, Treatments—red dots for individual data values and grey bars for the mean.

The roles of NR4A1 and NR4A2 in mediating the downregulation of MerTK were assessed in human and mouse colon cancer cell lines by RNA interference. Several NR4A1-targeting siRNAs and NR4A2-targeting antisense oligos (Figure 2A) were examined, and the most active oligos, siRNA N1-C and antisense oligo N2-A, were used to investigate the roles of NR4A1 and NR4A2 in the regulation of MerTK expression (Figure 2B). Ongoing studies show that individual knockdowns of NR4A1 or NR4A2 can result in downregulation of both receptors and that this cross-receptor regulation is cell context-dependent [38]. In human colon cancer cell lines, knockdown of NR4A1 decreased the expression of MerTK, NR4A1, and NR4A2 in all three cell lines (Figure 2B). Knockdown of NR4A2 decreased the expression of MerTK, NR4A2, and NR4A1 in SW480 and HCT116 cells. Some variability was also observed in mouse CT26 cells, as NR4A1 knockdown did not affect NR4A2 protein levels (Figure 2B). Furthermore, knockdown of both NR4A1 and NR4A2 resulted in a synergistic decrease in MerTK protein expression. Thus, knockdown of NR4A1 and/or NR4A2 in human and mouse colon cancer cells decreased MerTK protein levels, and the quantification of these results is summarized in Figure 2C. In addition, treatment of representative human (SW480) and mouse (CT26) colon cancer cells with DIM-3,5-Cl_2_ (Figure 2D) and knockdown of NR4A1 (Figure 2E) and NR4A2 (Figure 2F) in SW480 and CT26 colon cancer cells significantly decreased MerTK mRNA expression.

**Figure 3:**
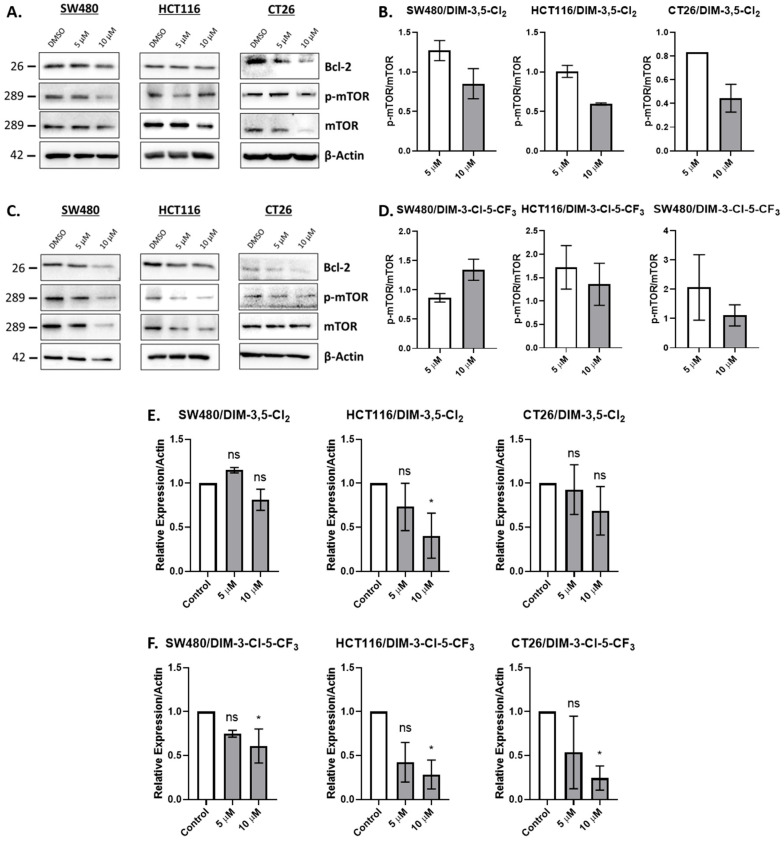
**Treatment of colon cancer cell lines with DIM-3,5 analogs decreases expression of selected downstream targets of MerTK signaling in a cell-context and dose-dependent manner.** (**A**) Colon cancer cells were treated with DIM-3,5-Cl_2_ for 18 h, and whole-cell lysates were analyzed using Western blotting and quantified relative to expression of β-actin. Lysates used in this Figure were the same lysates generated for data presented in Figure 1. (**B**) p-mTOR/mTOR ratios of the 5 μM and 10 μM dosages of DIM-3,5-Cl_2_ were quantified in relation to DMSO control (set as 1). (**C**) Colon cancer cells were treated with DIM-3-Cl-5-CF_3_ for 18 h, and whole-cell lysates were analyzed using western blotting and quantified relative to expression of β-actin. Lysates used in this Figure were the same lysates generated for data presented in Figure 1. (**D**) p-mTOR/mTOR ratios of the 5 μM and 10 μM dosages of DIM-3-Cl-5-CF_3_ were quantified in relation to DMSO control (set as 1). Expression of Bcl-2 in all three cell lines was quantified relative to expression of β-actin after treatment with (**E**) DIM-3,5-Cl_2_ and (**F**) DIM-3-Cl-5-CF_3_. Data is presented as mean ± SD of three biological replicates as described in Section 2.3. The significance levels are defined as follows: (*) for *p* < 0.05, and (ns) for *p* > 0.05.

Previous studies have shown that NR4A1 deficiency or treatment with bis-indole-derived NR4A1 and NR4A2 ligands decreased Bcl-2 expression [39,40,41] and inhibited mTOR signaling via induction of sestrin2 [39,40,41]. These responses were cancer cell and context-dependent [39,40,41,42]. MerTK also regulates the expression of mTOR and Bcl-2 in cancer cells [43,44]. Therefore, we investigated a possible role for MerTK in mediating NR4A1-dependent effects on phosphor-mTOR (p-mTOR), mTOR, and Bcl-2 in colon cancer cells treated with DIM-3,5-CI_2_ (Figure 3A, B) and DIM-3-CI-5-CF_3_ (Figure 3C, D), and ratios of p-mTOR/mTOR were assessed (Control: 5 μM: 10 μM). DIM-3,5-CI_2_ treatment resulted in a decrease in p-mTOR/mTOR ratios (SW480–1:1.2:0.85; HCT116–1:0.96:0.74; CT26–1:0.89:0.59), indicating that treatment with this compound decreased mTOR activity. However, treatment with DIM-3-CI-5-CF_3_ resulted in a cell context-dependent variability in mTOR activity (SW480–1:0.86:1.5; HCT116–1:1.9:1.7; CT26–1:1.6:1.3). The effects of DIM-3,5-CI_2_ (Figure 3E) and DIM-3-CI-5-CF_3_ treatments (Figure 3F) on Bcl-2 expression were also characterized. While DIM-3,5-CI_2_ treatment only resulted in a significant decrease in Bcl-2 expression in the human HCT116 colon cancer cell line, DIM-3-CI-5-CF_3_ treatment resulted in a significant decrease in Bcl-2 expression in all three cell lines. These data indicate that treatment with DIM-3,5-CI_2_ and DIM-3-CI-5-CF_3_ exhibited a dose-dependent and cell context-dependent variability in their effects on downstream NR4A1 and NR4A2 targets. Thus, DIM-3,5 analogs downregulated MerTK gene transcription in colon cancer cells, which was paralleled by decreased expression of downstream MerTK-regulated Bcl-2 and p-mTOR. However, the concentration-dependent differences in these responses indicated a possible role for MerTK in only those cells in which DIM-3,5 analogs decrease Bcl-2 or p-mTOR/mTOR ratios.

**Figure 4:**
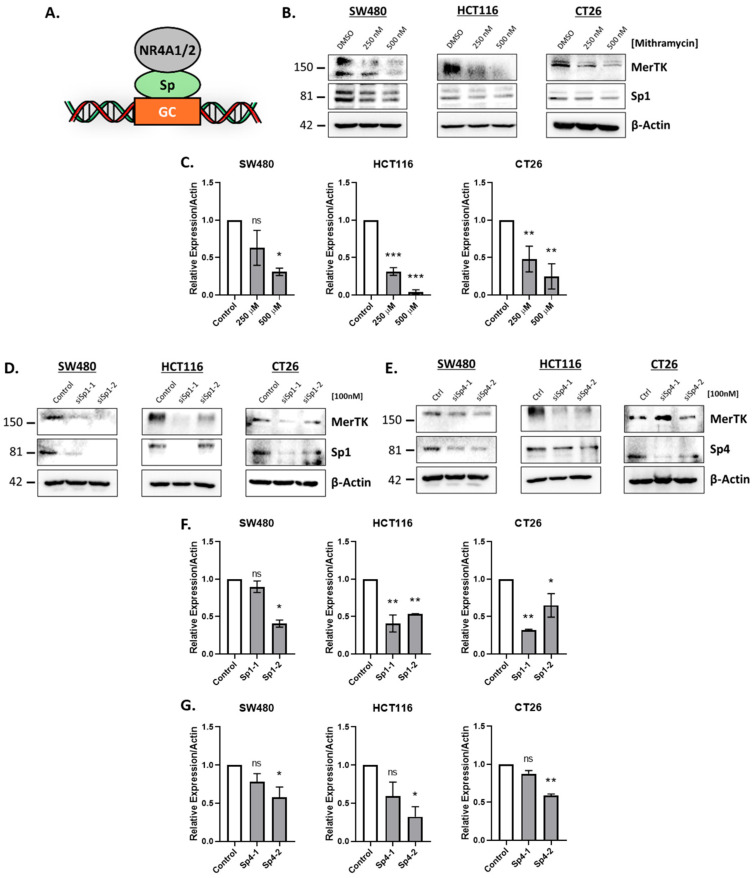
**Treatment with mithramycin**, an Sp1 inhibitor, **and Sp knockdown decreased MerTK expression in colon cancer cell lines.** (**A**) Structure of NR4A1/2:Sp complex. (**B**) SW480, HCT116, and CT26 cell lines were treated with the known Sp1 inhibitor, Mithramycin, and DMSO (control) for 18 h. (**C**) Whole-cell lysates were analyzed for MerTK expression by Western blotting and quantified relative to expression of β-actin. SW480, HCT116, and CT26 colon cancer cell lines were transfected with oligonucleotides targeting (**D**) Sp1 and (**E**) Sp4, and whole-cell lysates were obtained after 72 h. (**F**,**G**) Expression of MerTK was analyzed by Western blotting relative to β-actin expression, and data are presented as mean ± SD of three biological replicates as described in Section 2.3. The significance levels are defined as follows: (*) for *p* < 0.05, (**) for *p* < 0.01, (***) for *p* < 0.001, and (ns) for *p* > 0.05.

Previous studies have identified GC-rich sites in the proximal region of the MerTK promoter that are important for Sp1-regulated expression of the MerTK gene [32]. It has also been demonstrated that NR4A1 and NR4A2 act as cofactors of Sp1- and Sp4-mediated gene transcription (Figure 4A) [44], and therefore, the role of these complexes in regulating MerTK expression was investigated in colon cancer cells by treatment with mithramycin and knockdown of Sp1 and Sp4 by RNA interference. Mithramycin interacts with and inhibits Sp1 binding to GC-rich sites [45]. Treatment with mithramycin significantly decreased MerTK expression in all three colon cancer cell lines in a dose-dependent manner (Figure 4B,C). Further, knockdown of Sp1 (Figure 4D,F) and Sp4 (Figure 4E,G) using RNA interference showed that loss of Sp1 and Sp4 decreased MerTK protein levels in SW480, HCT116, and CT26 cells. Surprisingly, knockdown of Sp3 (Supplemental Figure S1A,B) also resulted in decreased expression of MerTK in all three cell lines. Previous studies have shown that Sp3-mediated gene expression is not coactivated by NR4A1 in pancreatic cancer cells [28]. These results, coupled with the observations that NR4A1 and NR4A2 knockdown also decreased MerTK levels in colon cancer cells, suggest that NR4A1 and NR4A2 act as ligand-dependent co-factors of Sp1, Sp3, and Sp4 to regulate MerTK expression in colon cancer cells.

**Figure 5:**
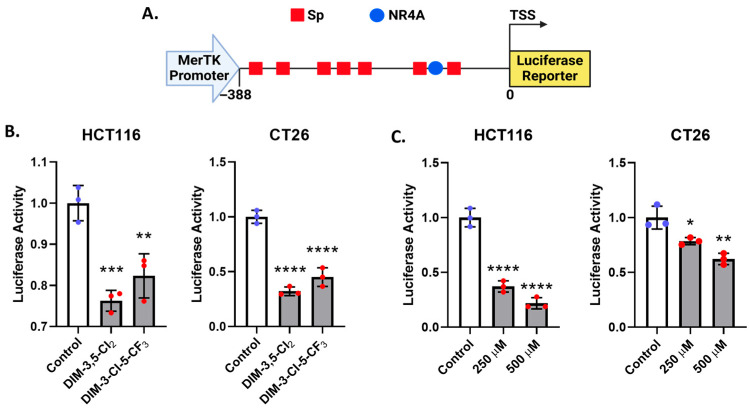
**DIM-3,5 compounds and mithramycin reduce MerTK-Luc activity, as shown by chromatin immunoprecipitation studies.** (**A**) Diagram of *MERTK* promoter indicating positions of Sp and NR4A binding sites as determined by JASPAR in silico promoter analysis. The construct (as described in Methods) was transfected into representative human HCT116 and mouse CT26 colon cancer cell lines, and transfected cells were treated with (**B**) 15 μM of the indicated DIM-3,5 ligands and (**C**) 250–500 nM of Mithramycin for 8 h. Luciferase activity was measured as indicated in Methods Section 2.5. Results are expressed as mean ± SD of three biological replicates. The significance levels are defined as follows: (*) for *p* < 0.05, (**) for *p* < 0.01, (***) for *p* < 0.001, (****) for *p* < 0.0001. Data is graphically represented as follows: Control—blue dots for individual data values and white bars for the mean, Treatments—red dots for individual data values and grey bars for the mean.

Using HCT116 and CT26 cells as models for investigating the effects of DIM-3,5 compounds and mithramycin on *MERTK* promoter activity, cells were transfected with a *MERTK*-luciferase construct containing the active GC-rich motifs (–388 to –177) in the *MERTK* gene promoter (Figure 5A). Both DIM-3,5-CI_2_ and DIM-3-CI-5-CF_3_ significantly decreased luciferase activity in HCT116 and CT26 cells (Figure 5B), transfected with the *MERTK*-luciferase construct. Further, treatment of HCT116 and CT26 cells with mithramycin also significantly decreased luciferase activity in a dose-dependent manner (Figure 5C).

**Figure 6:**
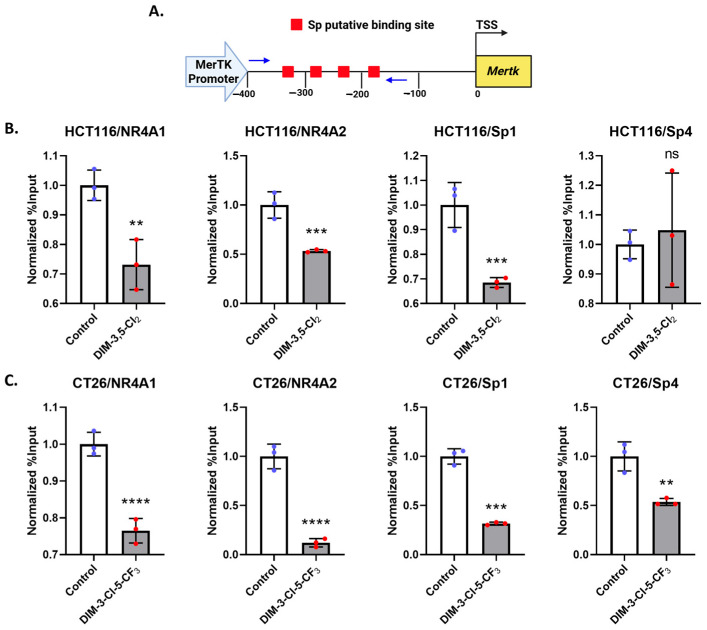
**ChIP analysis of binding to the MerTK promoter.** The primers used for the ChIP assay (**A**) on the human (–333 to –328/–287 to –282) and mouse (–234 to –229/–182 to –177) *MERTK* promoter. (**B**) The ChIP assay and subsequent analysis of binding to the human (HCT116) MerTK promoter showed that NR4A1, NR4A2, Sp1, and Sp4 were bound to the promoter and with the exception of Sp4 were decreased by treatment with DIM-3,5-Cl_2_. (**C**) Binding of NR4A1, NR4A2, Sp1 and Sp4 to the mouse (CT26) *MERTK* promoter was also detected and binding decreased after treatment DIM-3-CI-5-CF_3_. Results are normalized to β-actin chromatin input using the 2^-ΔΔ-CT^ method and expressed as means ± SD for three biological replicates as described in Section 2.6. The significance levels are defined as follows: (**) for *p* < 0.01, (***) for *p* < 0.001, (****) for *p* < 0.0001, and (ns) for *p* > 0.05. Data is graphically represented as follows: Control—blue dots for individual data values and white bars for the mean, Treatments—red dots for individual data values and grey bars for the mean.

The roles of NR4A1/Sp and NR4A2/Sp were further investigated in a ChIP assay in which primers were designed to determine interactions of NR4A1, NR4A2, and Sp TFs with the “active” GC-rich (Sp binding) sites in the *MERTK* promoter in both the human HCT116 and mouse CT26 cell lines (Figure 6A). The assay detected NR4A1, NR4A2, Sp1, and Sp4 binding to the human *MERTK* promoter in HCT116 cells (Figure 6B). After treatment with the inverse agonist DIM-3,5-Cl_2_ (15 μM), interactions of NR4A1, NR4A2, Sp1, and Sp4 with the proximal GC-rich region of the *MERTK* promoter significantly decreased. The assay was also performed on mouse CT26 cells, and binding of NR4A1, NR4A2, Sp1, and Sp4 to the mouse *MERTK* promoter was also detected (Figure 6C). After treatment with the inverse agonist DIM-3-Cl-5-CF_3_ (15 μM), interactions of NR4A1, NR4A2, Sp1, and Sp4 with the proximal GC-rich region of the *MERTK* promoter significantly decreased. Interestingly, binding of Sp3 to the mouse *MERTK* promoter was also decreased after DIM-3-Cl-5-CF_3_ (15 μM) treatment (Supplemental Figure S1C). These observations, coupled with the Sp and NR4A1 knockdown results and results obtained with transfection of the *MERTK*-luciferase construct, are consistent with a role for the NR4A/Sp complex in mediating the transcriptional regulation of MerTK.

**Figure 7:**
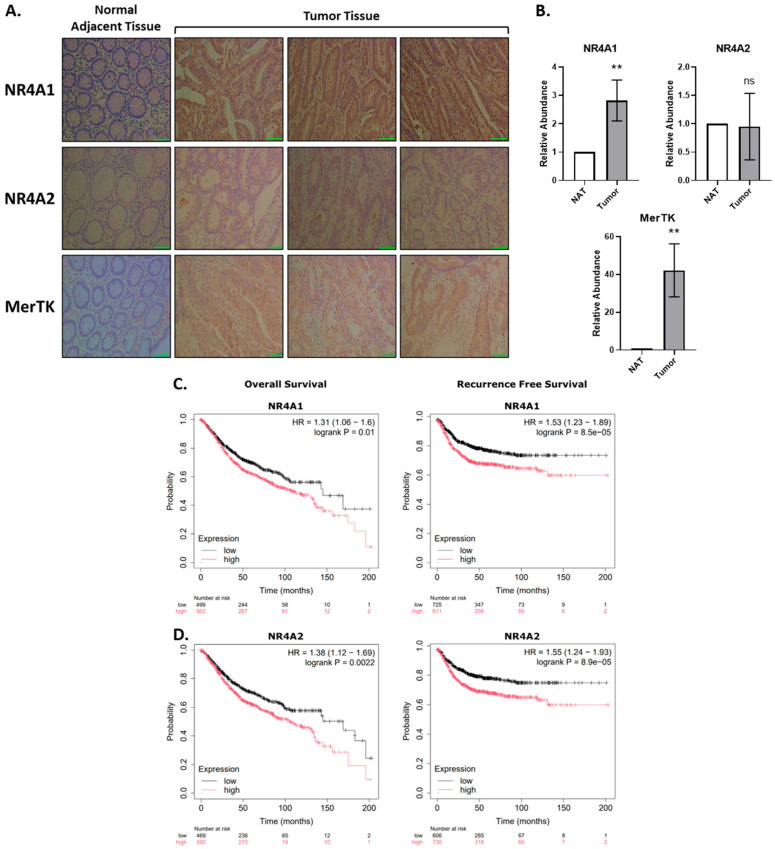
**Immunohistochemical analysis of NR4A1, NR4A2, and MerTK expression in colon cancer tissues.** (**A**) Representative immunohistochemical staining of NR4A1, NR4A2, and MerTK in colon cancer tissues and matched normal adjacent tissues (NAT) obtained from the same patients (n = 5). Brown staining indicates positive expression. Images were captured at 20× magnification; scale bar = 100 µm. (**B**) Quantification of NR4A1, NR4A2, and MerTK expression based on the percentage of DAB-positive area measured using ImageJ. For each sample, five random microscopic fields were analyzed and averaged to generate a single value per patient. Data are presented as mean ± SD of five independent patients (n = 5). Statistical significance is indicated as described in Section 2.9. Kaplan–Meier survival analyses showing the correlation between high and low expression levels of (**C**) NR4A1 and (**D**) NR4A2 with overall and recurrence-free survival in colon adenocarcinoma patients. Significance values for the survival analysis are indicated in the top-right corner of each graph and as follows: (**C**) NR4A1 Overall Survival—0.01, NR4A1 Recurrence Free Survival—8.5 × 10^−5^, (**D**) NR4A2 Overall Survival—0.0022, NR4A1 Recurrence Free Survival—8.9 × 10^−5^. (**) for *p* < 0.01, and (ns) for *p* > 0.05.

In addition, several studies have indicated that MerTK is overexpressed in colorectal cancer, making it an attractive therapeutic target [10,11]. Consistent with these reports, immunohistochemical analysis of tumor tissues and matched normal adjacent tissues (NAT) from colorectal cancer patients (n = 5) demonstrated increased expression of NR4A1 and MerTK in tumor tissues compared to NAT (Figure 7A,B). Quantification based on the percentage of DAB-positive area revealed higher staining intensity in tumor samples; however, given the semi-quantitative nature of IHC, these data are presented as relative differences rather than fold-change values. In contrast, NR4A2 expression was variable across tumor samples and did not show a consistent difference compared to matched normal tissues (Figure 7A,B). Furthermore, Kaplan–Meier survival analysis indicates that higher expression of both NR4A1 and NR4A2 is associated with reduced overall and recurrence-free survival among patients with colon adenocarcinoma (Figure 7C,D).

Together, these findings support a role for the NR4A/Sp regulatory axis in controlling MerTK expression in colon cancer and suggest that MerTK may represent a potential therapeutic target. In this context, DIM-3,5 analogs, which function as NR4A1/2 inverse agonists, may inhibit MerTK expression by disrupting NR4A/Sp-mediated transcriptional activity.

## 4. Discussion/Conclusions

MerTK is a member of the TAM family of tyrosine kinases that plays an important pro-oncogenic role in multiple cancers, including colon cancer, and is a negative prognostic factor for colon cancer patients [5,6,7,8,9,10,11]. NR4A1 and NR4A2 are also pro-oncogenic factors in most solid tumors, and their knockdown by RNA interference or treatment with DIM-3,5 compounds that bind both NR4A1 and NR4A2 inhibit the pro-oncogenic activities of both receptors [27]. MerTK is also a cancer therapeutic target, and neutralizing antibodies, small-molecule kinase inhibitors, and protein degraders are being developed to block MerTK-mediated pro-oncogenic activities [19,20,21,22,23,24,25,26]. Thus, MerTK, NR4A1, and NR4A2 are druggable targets for cancer chemotherapy. A recent study in non-small cell lung cancer cells showed that the flavonoid luteolin inhibited cell growth and downregulated MerTK levels [3]. Since studies in this laboratory have recently reported that flavonoids such as luteolin, quercetin, and kaempferol bind to NR4A1 and inhibit NR4A1-dependent pro-oncogenic pathways/genes [34,46], this suggests that other NR4A1 or NR4A2 ligands may also target the downregulation of MerTK. Moreover, since MerTK has previously been characterized as an Sp1-regulated gene [32,47], we hypothesized that MerTK may be an NR4A1/2/Sp-regulated gene that can be directly targeted by DIM-3,5 dual NR4A1/2 ligands acting as inverse agonists in cancer cells.

Treatment of human and mouse colon cancer cells with DIM-3,5-CI_2_ and DIM-3-CI-5-CF_3_ decreased expression of MerTK, and this was accompanied by the cell context-dependent and dose-dependent downregulation of some MerTK-regulated responses, including Bcl-2 and p-mTOR. However, the results suggested that the effects of NR4A1/2 ligands on these responses were attributable not only to MerTK downregulation but also to other NR4A1/2-regulated pathways, and that these pathways were both ligand- and cell context-dependent [39,40,41].

Several studies have shown that knockdown of NR4A1 or NR4A2 decreases expression of receptor-regulated genes, and knockdown of Sp1 or Sp4 also decreases expression of some of the same genes, which include survivin, PAX3-FOX01, PD-L1, β1- and β3-integrins, TWIST1, and G9a [28,29,30,31,48,49]. These data suggest that NR4A1 and NR4A2 may act as ligand-dependent cofactors of some Sp-regulated genes. Similar results have also been observed using mithramycin, which blocks Sp interactions with GC-rich promoter regions [45]. Moreover, a recent study has shown that both NR4A1 and NR4A2 interact with Sp binding sites on the TWIST1 promoter [48]. It is possible that these receptors may act as heterodimers since it has been shown that NR4A1 and NR4A2 form heterodimers [50]. Results obtained in this study showed that knockdown of NR4A1, NR4A2, and Sp1, Sp3, and Sp4 or treatment of colon cancer cells with mithramycin and DIM-3,5 ligands decreased MerTK protein and mRNA expression. Both ChIP and promoter (*MERTK*)-luciferase assays supported a role for NR4A1 and NR4A2 acting as cofactors in NR4A/Sp-mediated expression of MerTK. In contrast to previous studies with NR4A1 and NR4A2, we have also shown that not only Sp1 and Sp4 but also Sp3 is involved in MerTK regulation. A previous report also showed that NR4A1 regulated MerTK in bone marrow-derived macrophages through interactions as a monomer with a nerve growth factor B response element (NBRE) in the intronic region of the *MERTK* promoter [51], and this was in contrast to what is observed in a colon cancer cell context.

Immunohistochemical analysis of tissues of colon cancer patients indicated that expression of NR4A1 and MerTK was significantly increased in tumor tissues relative to adjacent normal tissues, indicating that both NR4A1 and MerTK are pro-oncogenic in colorectal cancer. In contrast, NR4A2 expression was variable in tumor tissues. In addition, Kaplan–Meier survival plot data suggest that high expression of NR4A1 and NR4A2 is associated with lower overall and recurrence-free survival in colorectal cancer patients. Together, these data suggest that NR4A1, NR4A2, and MerTK are possible targets for treating colorectal cancer.

MerTK plays a critical role as a pro-oncogenic factor in carcinogenesis, and this kinase also influences the parallel immune systems. There are ongoing studies focusing on targeting this gene. The advantages of using DIM-3,5 compounds that decrease transcriptional regulation of MerTK include their inhibition of NR4A1/2-regulated cancer cell proliferation, survival, migration, and invasion pathways/genes and activation of CD8^+^ T-cells [27]. In addition, these data, along with previous studies, indicate that targeting the NR4A/Sp complexes decreases expression of several oncogenic factors. In summary, this study shows that the NR4A1/2/Sp(1-3) complex regulates MerTK expression in colon cancer cells. DIM-3,5 dual NR4A1 and NR4A2 ligands act as inverse agonists to downregulate MerTK expression. The mechanism involves ligand-dependent inactivation of both NR4A1 and NR4A2, which act as cofactors of Sp-regulated expression of MerTK. DIM-3,5-mediated inhibition of MerTK gene expression provides a novel transcription control-based approach for inhibiting this kinase in colon cancer cells, and this is currently being investigated in other solid tumor-derived cancers.

## Data Availability

The original contributions presented in this study are included in the article/Supplementary Materials. Further inquiries can be directed at the corresponding author.

## Supplementary Materials

The following supporting information can be downloaded at: https://www.mdpi.com/article/10.3390/cancers18121993/s1, Figure S1. Role of Sp3 in regulation of MerTK. Analysis of whole cell lysates after knockdown of Sp3 in SW480, HCT116 and CT26 cells showed that MerTK was also decreased (A,B), and Sp3 interactions with the CT26 promoter (GC-rich region) after treatment were also decreased (C) in a ChIP assay. Results (B and C) are means ± SD for three replicated determinations, and significant (*p* < 0.05) decreases compared to controls are indicated.; Table S1: Antibodies used for Western blotting.; Table S2: Antibodies used for ChIP.; Table S3: qPCR Primers (mRNA Expression); Table S4: ChIP Primers; Table S5: Antibodies used for Immunohistochemistry. File S1: Full Blots for Western Images.

## Abbreviations

The following abbreviations are used in this manuscript:

CDIMs	bis-indole derived compounds
DIM-3,5	1,1-bis(3′-indolyl)-1-(3,5-disubstitutedphenyl)methane
DMSO	dimethylsulfoxide
FBS	fetal bovine serum
Luc	luciferase
NR4A1	nuclear receptor 4A1
PCR	polymerase chain reaction
PROS1	vitamin K dependent protein S1
RTK	receptor tyrosine kinase
Sp	specificity protein
