# Supplementary material for: MerTK Is Regulated by Orphan Nuclear Receptor 4A1 (NR4A1) and NR4A2 in Colon Cancer Cells
Source: Cancers (Basel). 2026 Jun 18;18(12):1993. doi: 10.3390/cancers18121993 (PMC13296424; doi:10.3390/cancers18121993)
Supplement: Supplementary file 1 [file cancers-18-01993-s001.zip › cancers-4362528-supplementary-english-6.16.pdf]

**Supplemental Materials**

**Supplemental Figure S1 ......................................................................... pg 2**

**Supplemental Table S1 ........................................................................... pg 3**

**Supplemental Table S2 ........................................................................... pg 3**

**Supplemental Table S3 ........................................................................... pg 3**

**Supplemental Table S4 ........................................................................... pg 3**

**Supplemental Table S5 ........................................................................... pg 4**

**Supplemental Figure:**

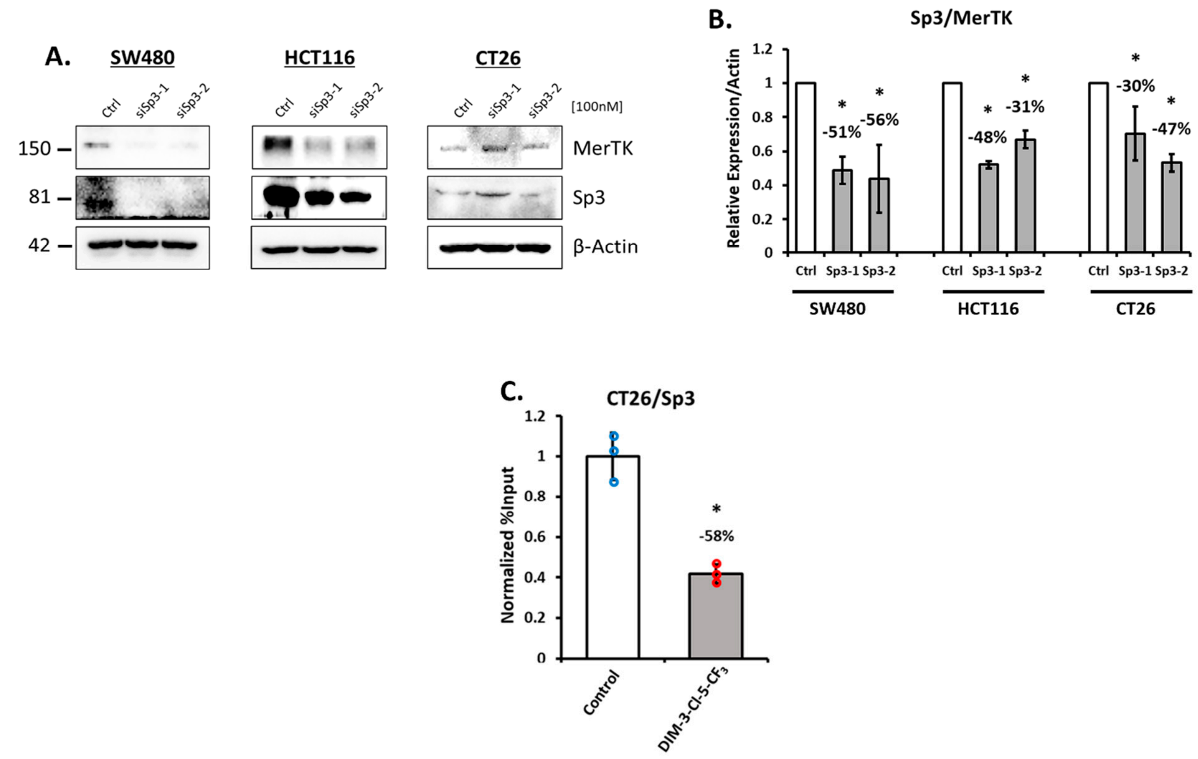


**Figure S1. Role of Sp3 in regulation of MerTK.** Analysis of whole cell lysates after knockdown of Sp3 in SW480, HCT116 and CT26 cells showed that MerTK was also decreased **(A,B),** and Sp3 interactions with the CT26 promoter (GC-rich region) after treatment were also decreased **(C)** in a ChIP assay. Results **(B and C)** are means ± SD for three replicated determinations, and significant ($p < 0.05$) decreases compared to controls are indicated.

**Supplemental Tables:**

**Supplemental Table S1.** Antibodies used for Western blotting.

| Antibody | Vendor | Catalog No. | WB Dilution |
|---|---|---|---|
| B-Actin | Sigma | A5316 | 1000 |
| MerTK (Human) | Cell Signaling | 4319 | 500 |
| MerTK (Mouse) | R&D Systems | AF591 | 500 |
| NR4A1 | Abcam | ab 283264 | 500 |
| NR4A2 | Santa Cruz | sc-376984 | 1000 |
| Sp1 | Santa Cruz | sc-17824 | 500 |
| Sp3 | Santa Cruz | sc-644 | 500 |
| Sp4 | Santa Cruz | sc-390124 | 500 |
| mTOR | Cell Signaling | 2972 | 500 |
| p-mTOR | Cell Signaling | 2971 | 500 |
| Bcl-2 | Cell Signaling | 4223 | 500 |

**Supplemental Table S2:** Antibodies used for ChIP

| Antibody | Vendor | Catalog No. | Amount (μg) |
|---|---|---|---|
| NR4A1 | Santa Cruz | sc-365113 X | 6 |
| NR4A2 | Santa Cruz | sc-376984 | 1000 |
| Sp1 | Santa Cruz | sc-17824 | 3 |
| Sp3 | Santa Cruz | sc-365220 X | 3 |
| Sp4 | Santa Cruz | sc-390124 | 3 |
| Mouse IgG | Santa Cruz | sc-2025 | 1.5 |

**Supplemental Table S3:** qPCR Primers (mRNA Expression)

| Species | Primer | Sequence |
|---|---|---|
| Human (SW480) | β-Actin | Forward: 5′ CACCATTGGCAATGAGCGGTTC 3′<br>Reverse: 5′ AGGTCTTTGCGGATGTCCACGT 5′ |
| | MerTK | Forward: 5′ CAGGAAGATGGGACCTCTCTGA 3′<br>Reverse: 5′ GGCTGAAGTCTTTCATGCACGC 3′ |
| Mouse (CT26) | β-Actin | Forward: 5′ AGTGTGACGTTGACATCCGTA 3′<br>Reverse: 5′ GCCAGAGCAGTAATCTCCTTC 5′ |
| | MerTK | Forward: 5′ ATCATCCTCGGCTGCTTCTGTG 3′<br>Reverse: 5′ ACGACCAGTTGGGAATCCTCCT 3′ |

**Supplemental Table S4:** ChIP Primers

| Species | Primer | Sequence |
|---|---|---|
| Mouse (CT26) | β-Actin | Forward: 5′ AGTGTGACGTTGACATCCGTA 3′<br>Reverse: 5′ GCCAGAGCAGTAATCTCCTTC 5′ |
| | MerTK | Forward: 5′ CGCTCTCCACTAAATTCGGC 3′<br>Reverse: 5′ AAATCTGGAAGGGAGGTGCT 3′ |

**Supplemental Table S5: Antibodies used for Immunohistochemistry**

| Antibody | Vendor | Catalog No. | WB Dilution |
|---|---|---|---|
| NR4A1 | ProteinTech | 2585-1-AP | 100 |
| NR4A2 | Santa Cruz | sc-376984 | 50 |
| MerTK | Cell Signaling | 4319 | 100 |
