# Supplementary material for: MerTK Is Regulated by Orphan Nuclear Receptor 4A1 (NR4A1) and NR4A2 in Colon Cancer Cells
Source: Cancers (Basel). 2026 Jun 18;18(12):1993. doi: 10.3390/cancers18121993 (PMC13296424; doi:10.3390/cancers18121993)
Supplement: Supplementary file 1 [file cancers-18-01993-s001.zip › File S1-Full Blots for Western Images.pdf]

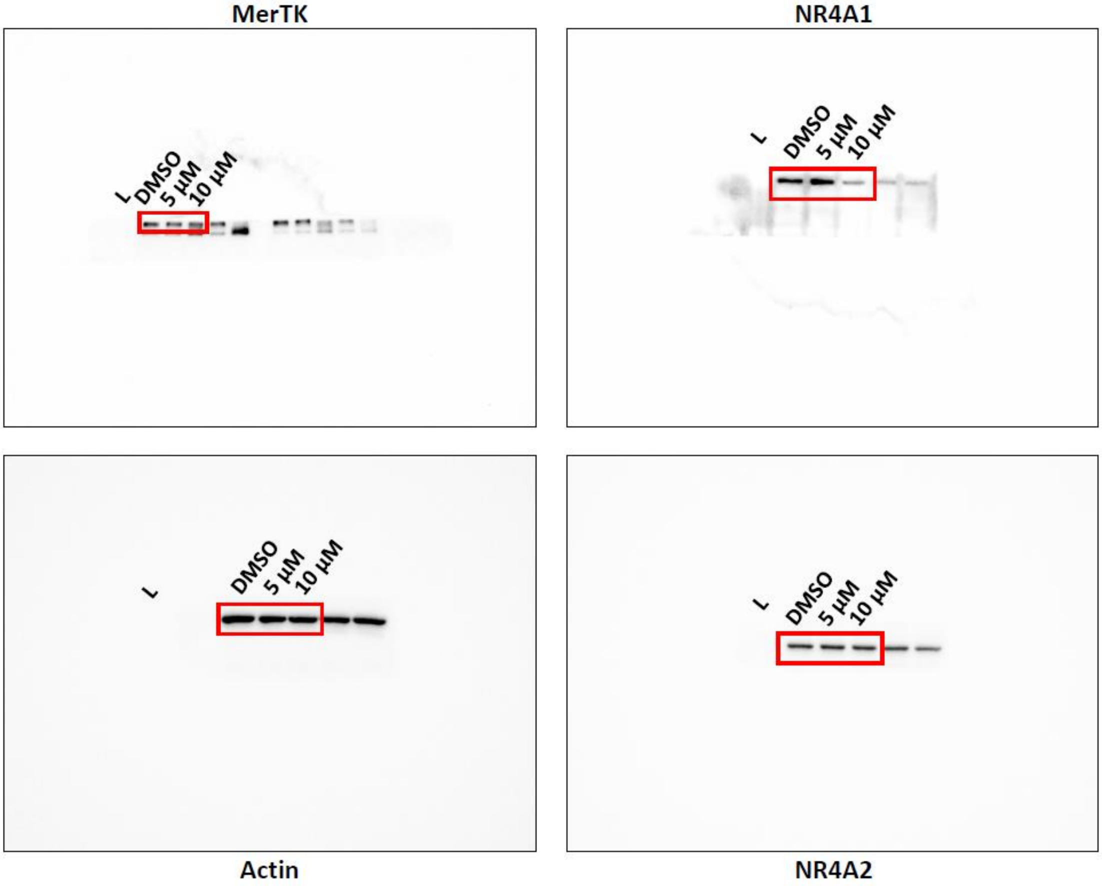

Figure S2: Whole western blot images in Figure 1B – SW480 (DIM-3,5-Cl2 Treatment). Drug treatment for SW480. Red boxes indicate the cropped sections displayed in the main figure. Full blot was cut prior to primary antibody incubation to detect multiple proteins in the same blot. Since NR4A1 and NR4A2 are of similar molecular weights, NR4A1 was imaged first, then blot was stripped and reimaged for NR4A2. MerTK was measured on a separate blot using the same samples on the same day due to errors in gel making.

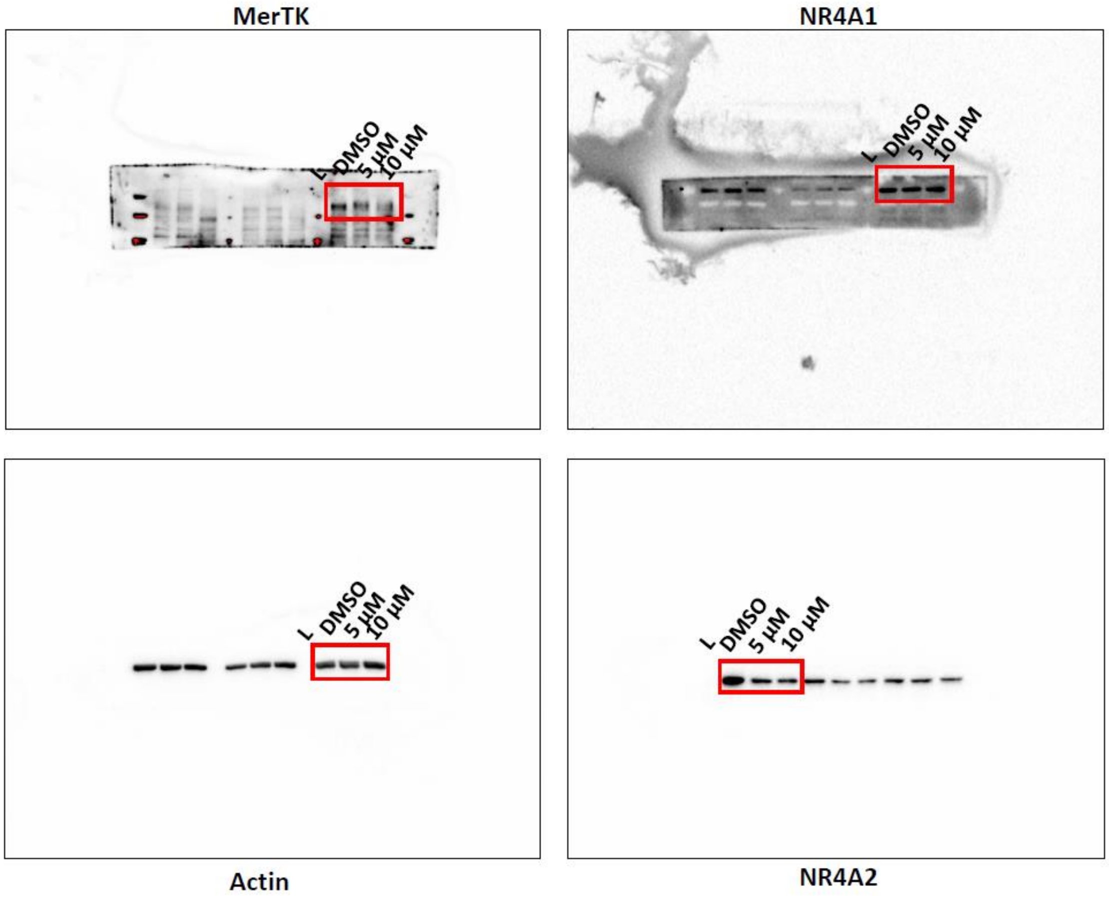

Figure S3: Whole western blot images in Figure 1B – HCT116 (DIM-3,5-Cl2 Treatment). Drug treatment for HCT116. Red boxes indicate the cropped sections displayed in the main figure. Full blot (for NR4A1, Actin and MerTK) was cut prior to primary antibody incubation to detect multiple proteins in the same blot. Since NR4A1 and NR4A2 are of similar molecular weights, NR4A2 was run and imaged on a separate blot (same samples, same loading amounts).

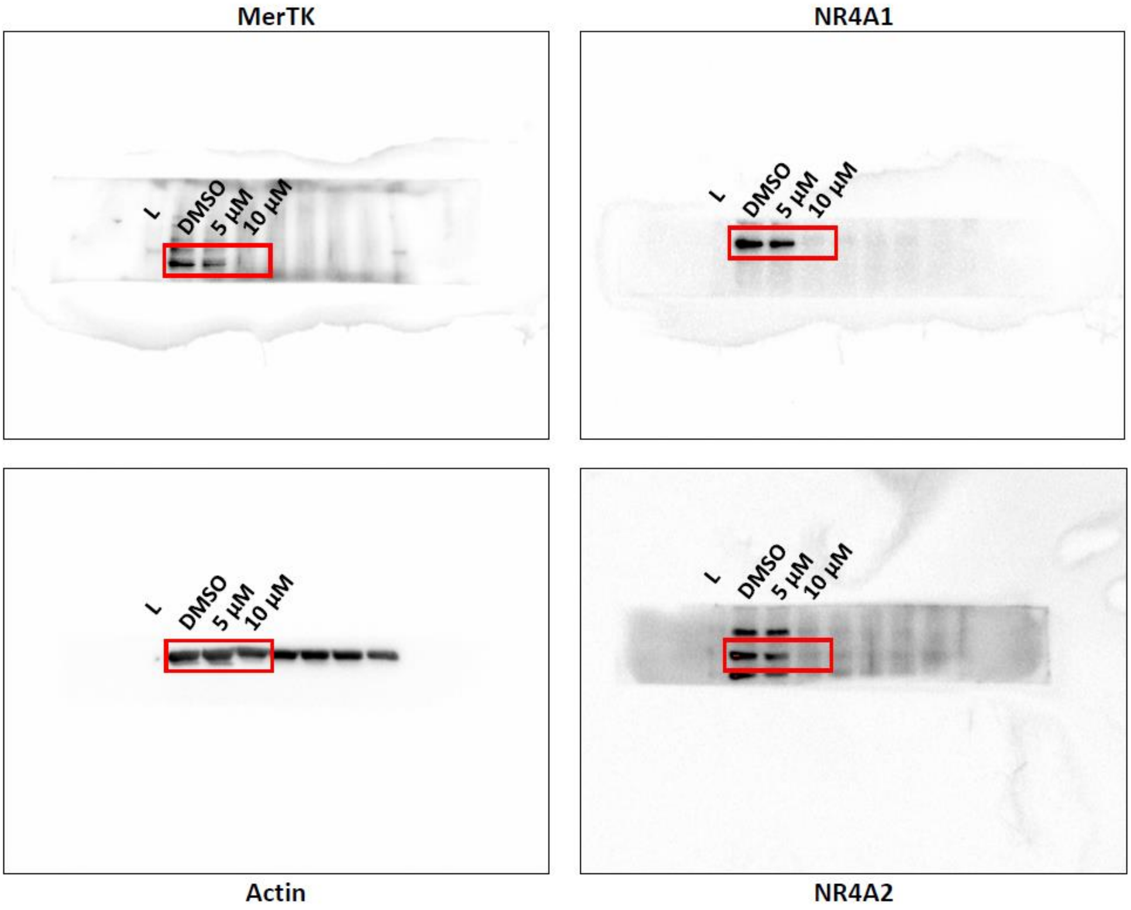

Figure S4: Whole western blot images in Figure 1B – CT26 (DIM-3,5-Cl2 Treatment). Drug treatment for CT26. Red boxes indicate the cropped sections displayed in the main figure. Full blot was cut prior to primary antibody incubation to detect multiple proteins in the same blot. Since NR4A1 and NR4A2 are of similar molecular weights, NR4A1 was imaged first, then blot was stripped and reimaged for NR4A2.

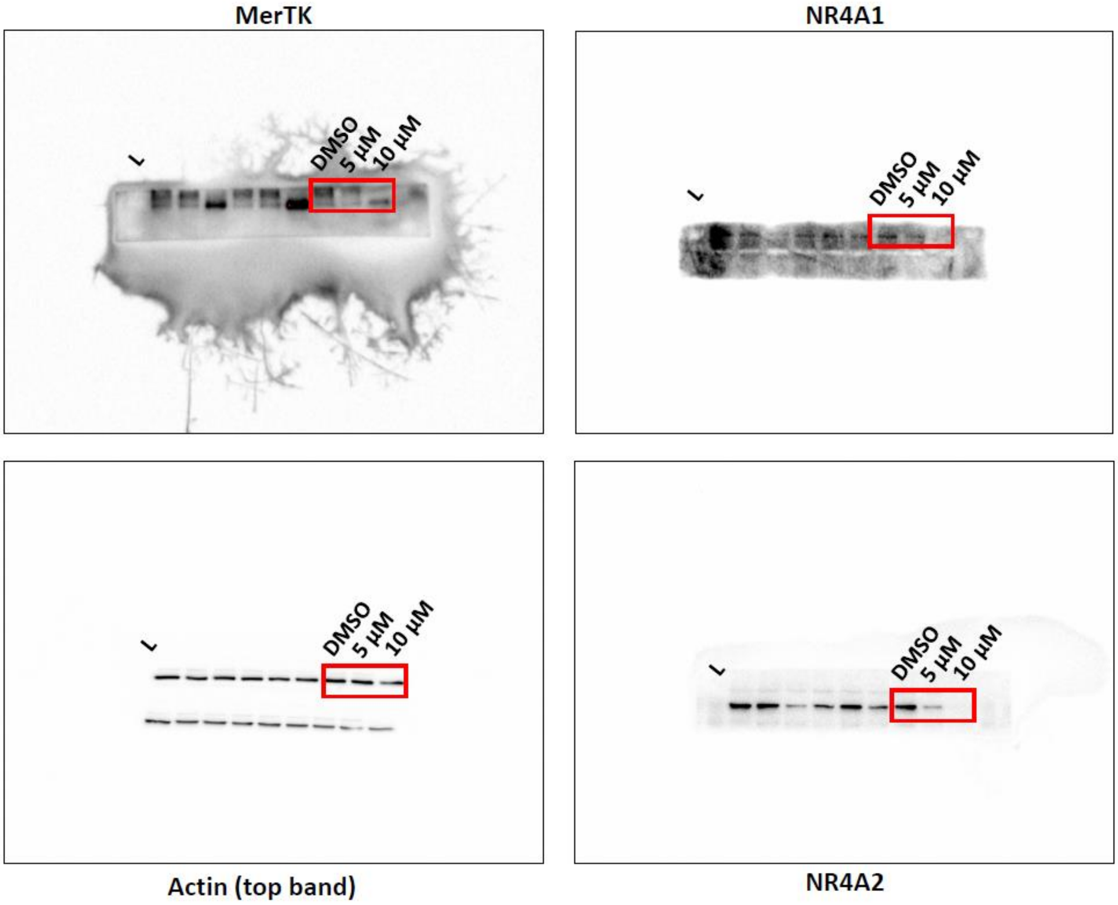

Figure S5: Whole western blot images in Figure 1C – SW480 (DIM-3-Cl-5-CF3 Treatment). Drug treatment for SW480. Red boxes indicate the cropped sections displayed in the main figure. Full blot was cut prior to primary antibody incubation to detect multiple proteins in the same blot. Since NR4A1 and NR4A2 are of similar molecular weights, NR4A1 was imaged first, then blot was stripped and reimaged for NR4A2.

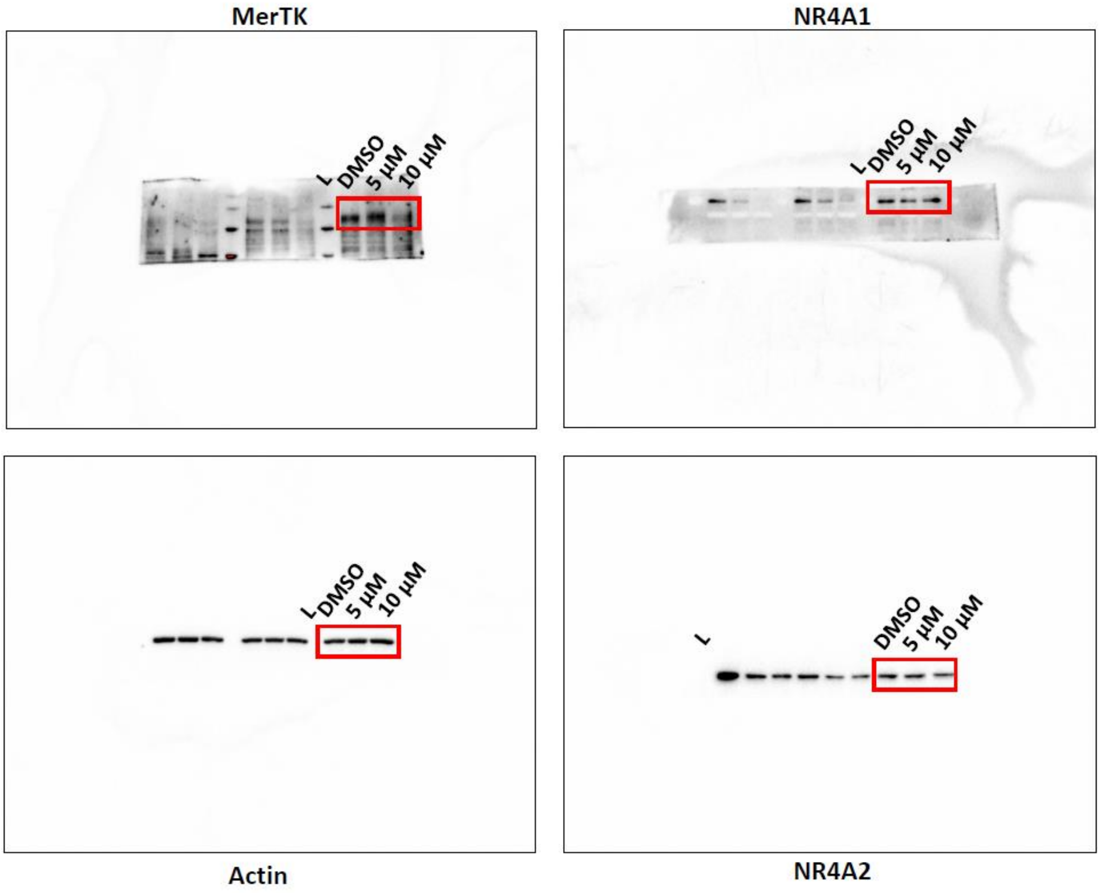

Figure S6: Whole western blot images in Figure 1C – HCT116 (DIM-3-Cl-5-CF3 Treatment). Drug treatment for HCT116. Red boxes indicate the cropped sections displayed in the main figure. Full blot (for NR4A1, Actin and MerTK) was cut prior to primary antibody incubation to detect multiple proteins in the same blot. Since NR4A1 and NR4A2 are of similar molecular weights, NR4A2 was run and imaged on a separate blot (same samples, same loading amounts)

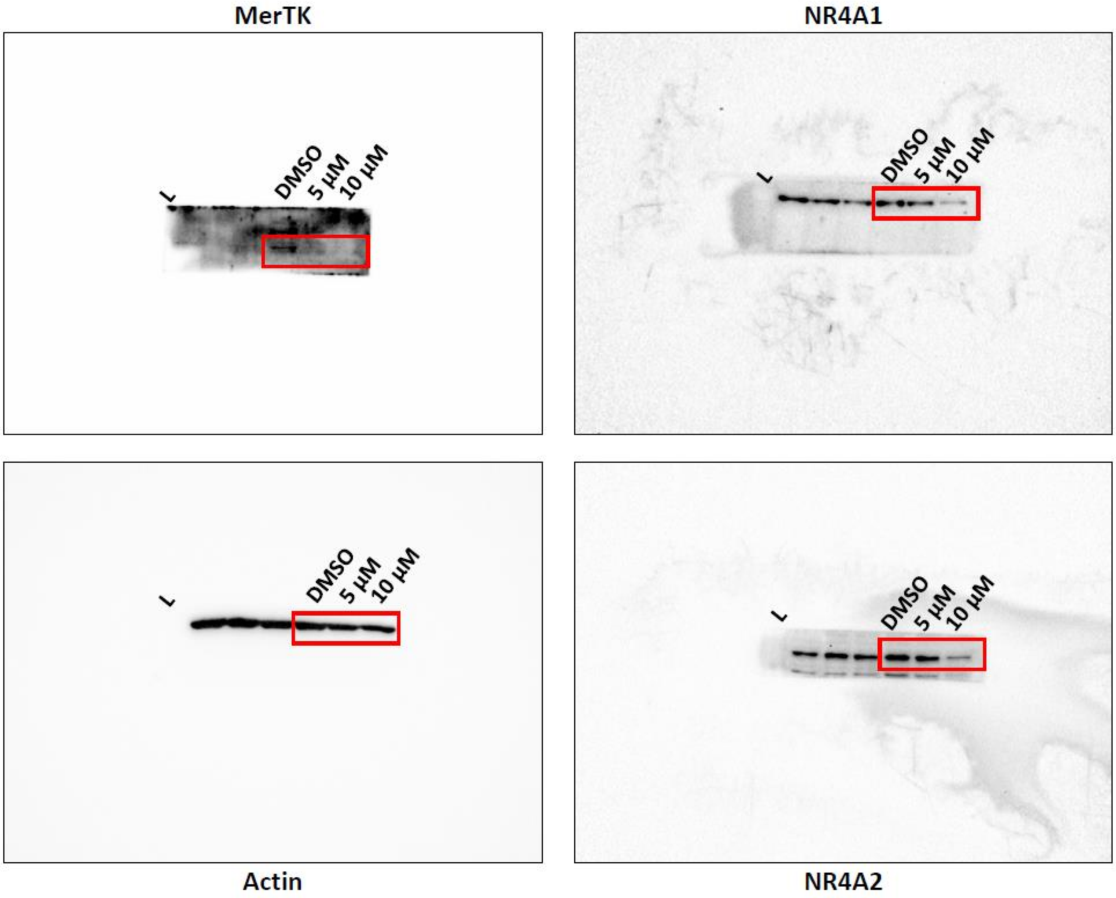

Figure S7: Whole western blot images in Figure 1C – CT26 (DIM-3-Cl-5-CF3 Treatment). Drug treatment for CT26. Red boxes indicate the cropped sections displayed in the main figure. Full blot was cut prior to primary antibody incubation to detect multiple proteins in the same blot. Since NR4A1 and NR4A2 are of similar molecular weights, NR4A1 was imaged first, then blot was stripped and reimaged for NR4A2.

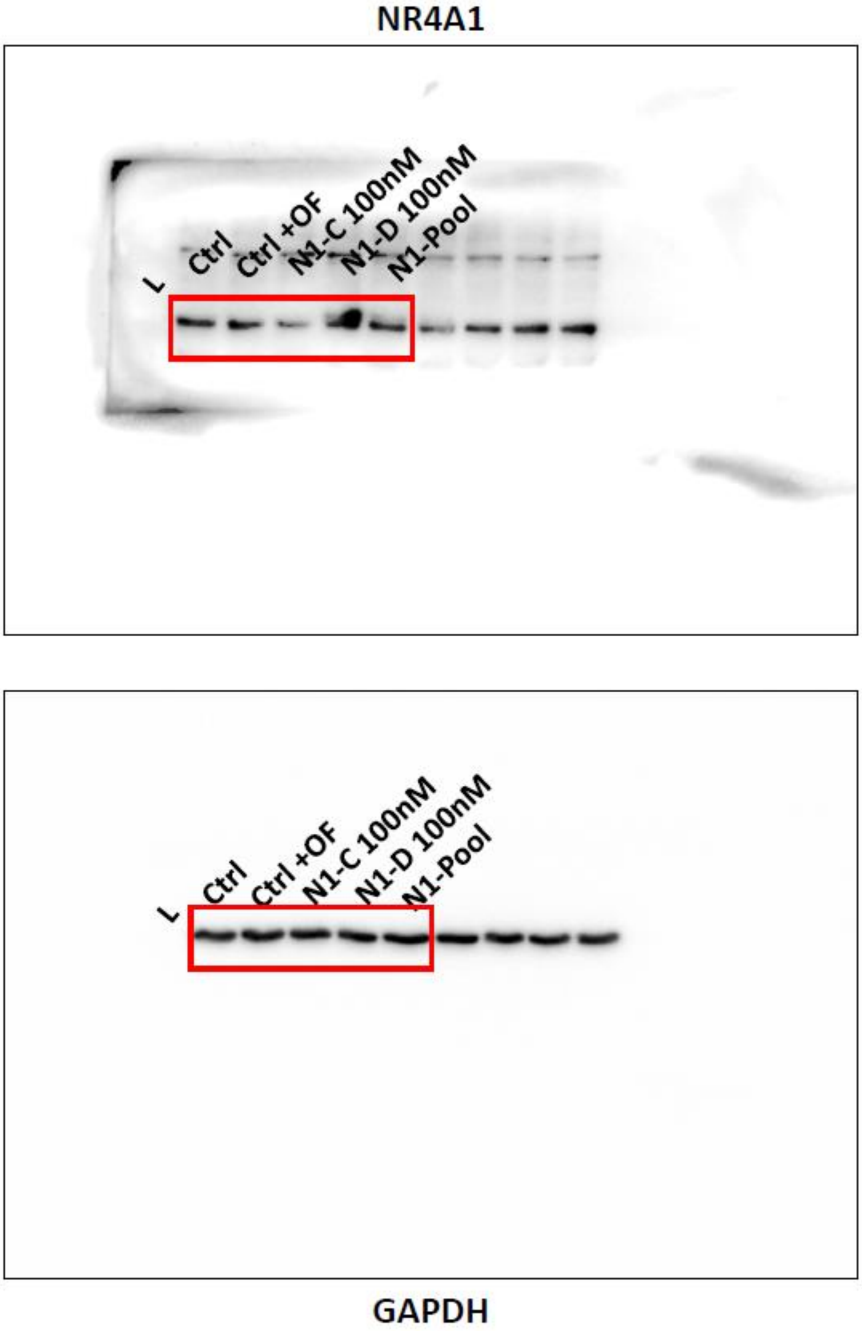

Figure S8: Whole western blot images in Figure 2A – SW480 (NR4A1 Knockdown Validation). Validation for the usage of siRNA N1-C for future experiments. Comparing three siRNAs, N1-C, N1-D and N1-pool (all at 100 nM final concentration) indicated that N1-C delivered the knockout and would be appropriate to use for future experiments.

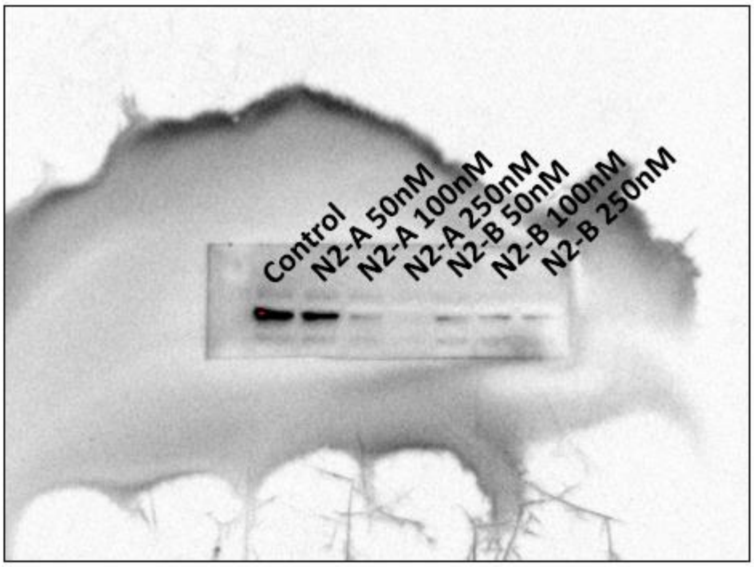

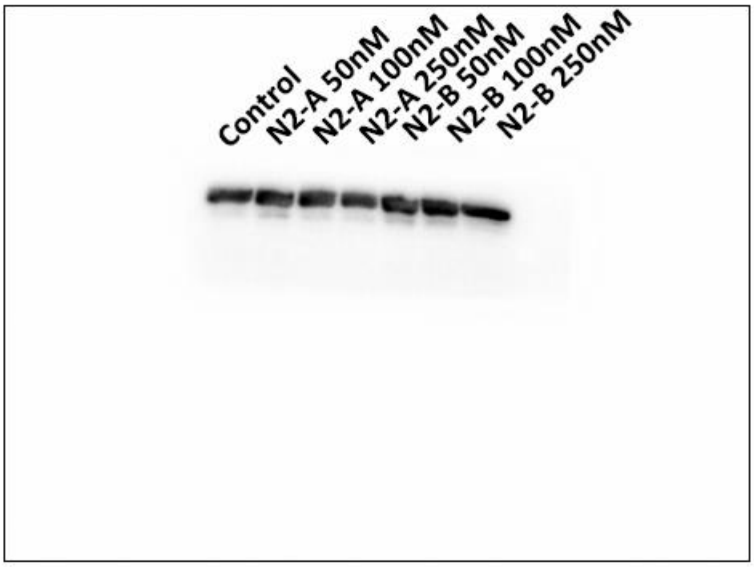

Actin

Figure S9: Whole western blot images in Figure 2B – SW480 (NR4A2 Knockdown Validation). Validation for the usage of Anti-sense RNA N2-A for future experiments. Comparing three siRNAs, N2-A and N2-B at varying concentrations indicated that N2-A delivered a dose-response verified knockout and would be appropriate to use for future experiments.

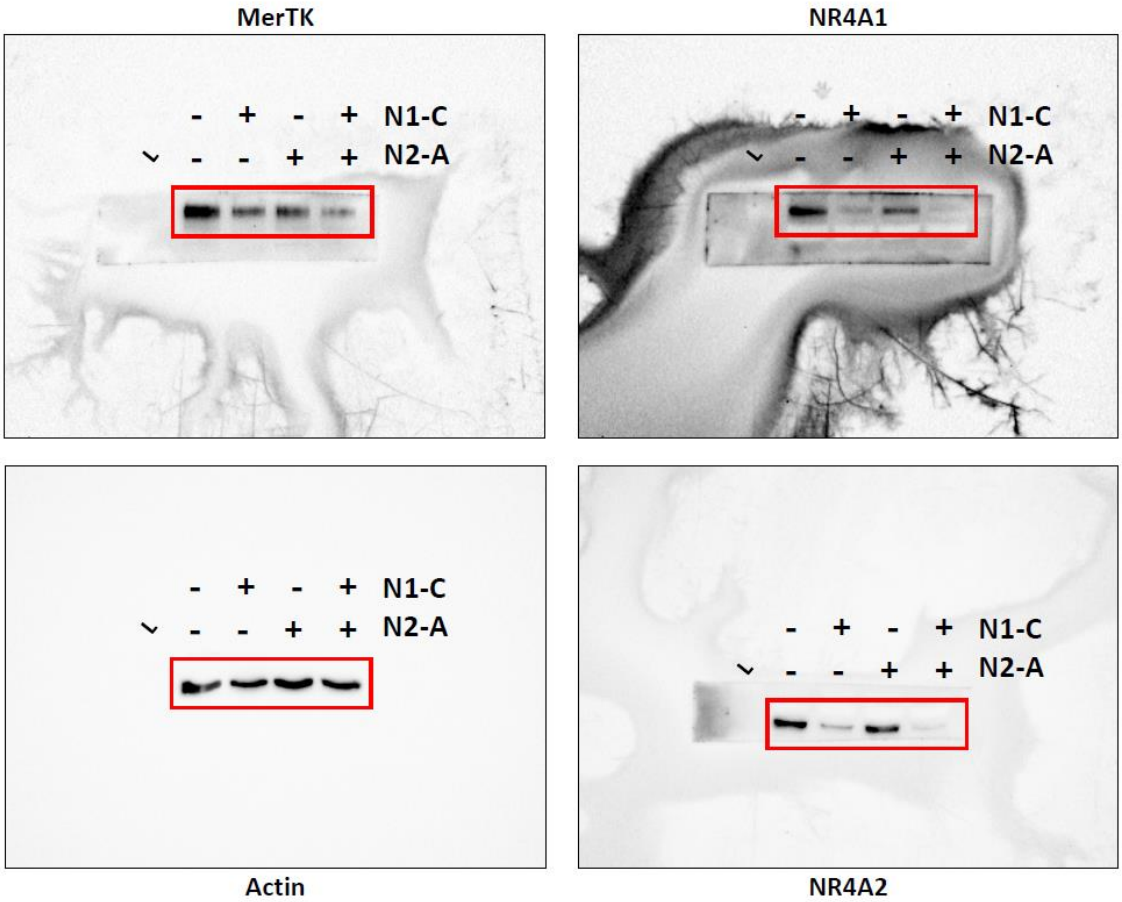

Figure S10: Whole western blot images in Figure 2C – SW480 (NR4A1/2 Knockdown). NR4A1/2 knockdown treatment for SW480. Red boxes indicate the cropped sections displayed in the main figure. Full blot was cut prior to primary antibody incubation to detect multiple proteins in the same blot. Since NR4A1 and NR4A2 are of similar molecular weights, NR4A1 was imaged first, then blot was stripped and reimaged for NR4A2.

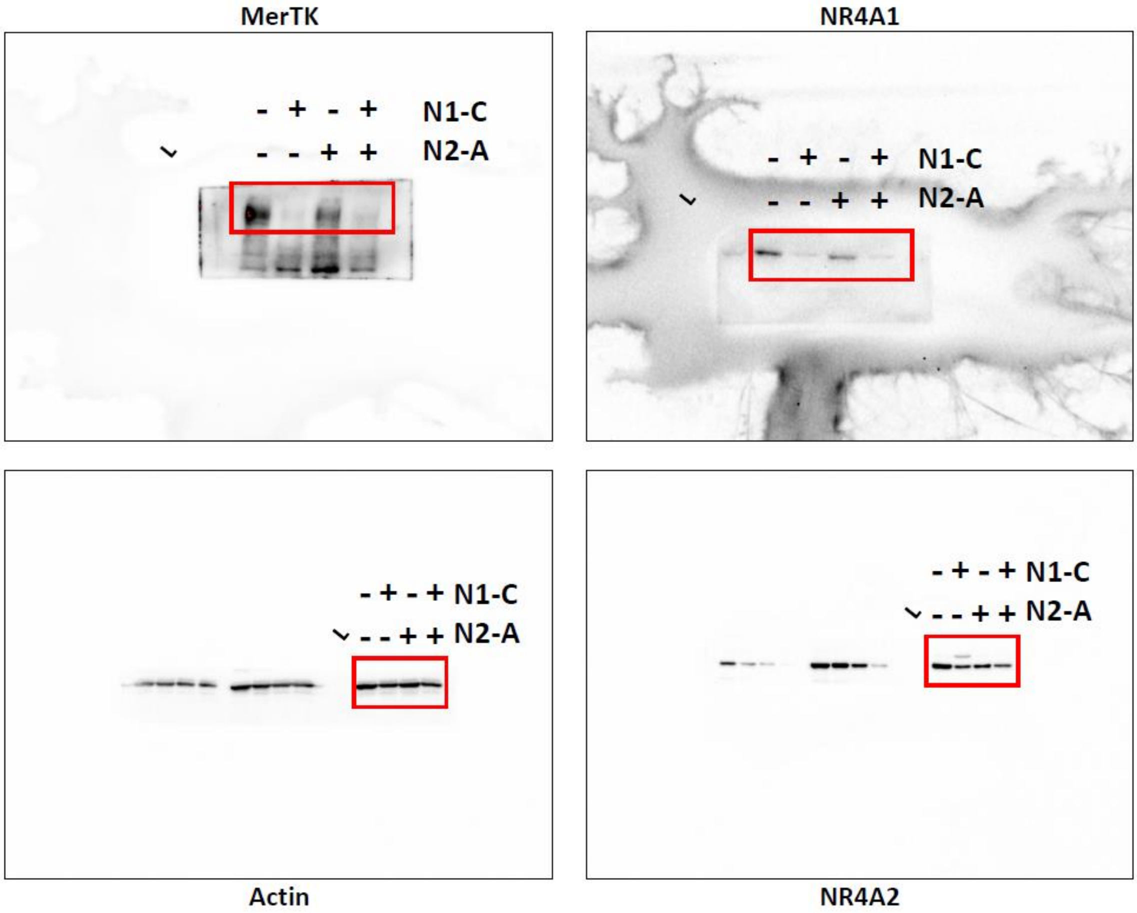

Figure S11: Whole western blot images in Figure 2C – HCT116 (NR4A1/2 Knockdown). NR4A1/2 knockdown treatment for HCT116. Red boxes indicate the cropped sections displayed in the main figure. Actin and NR4A2 were run on the same blot with other samples. NR4A1 and MerTK, using the same samples, were run on a separate blot.

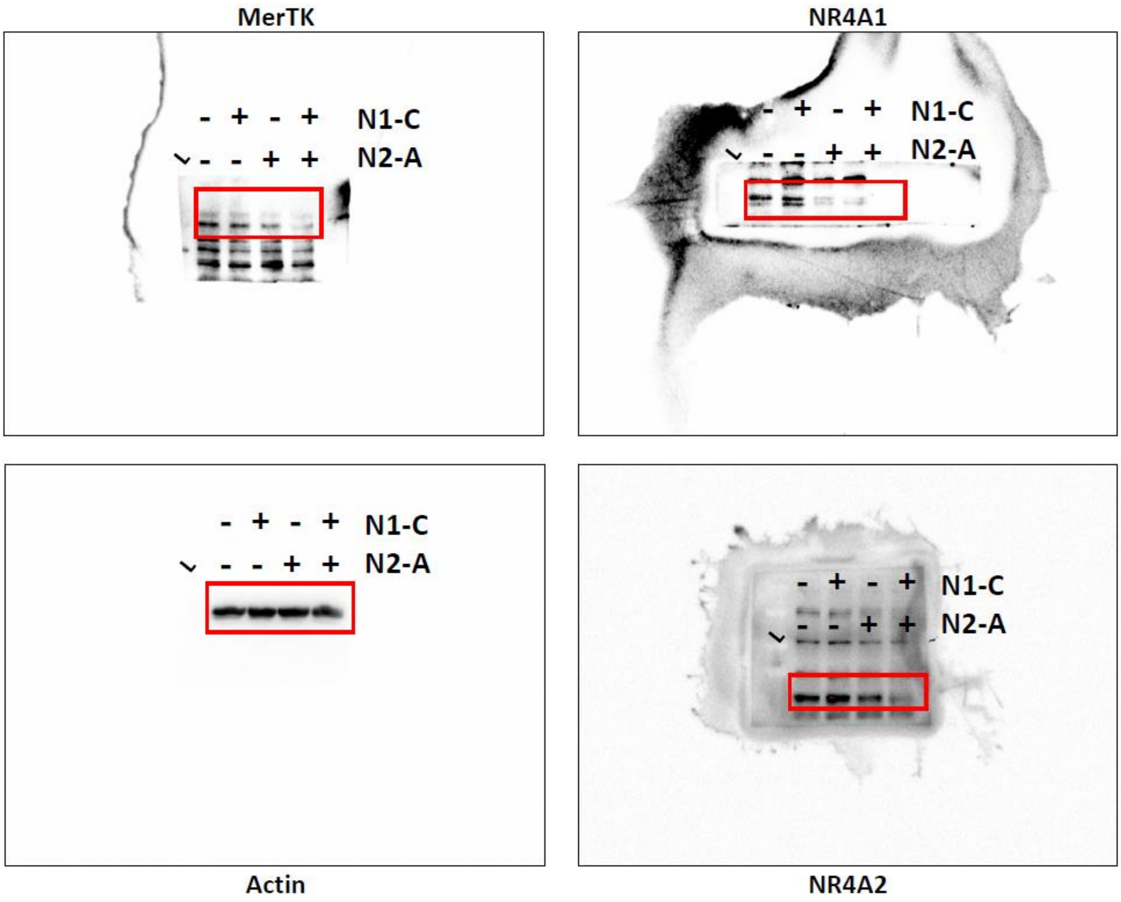

Figure S12: Whole western blot images in Figure 2C – CT26 (NR4A1/2 Knockdown). NR4A1/2 knockdown treatment for CT26. Red boxes indicate the cropped sections displayed in the main figure. Actin and NR4A2 were run on the same blot with other samples. NR4A1 and MerTK, using the same samples, were run on a separate blots.

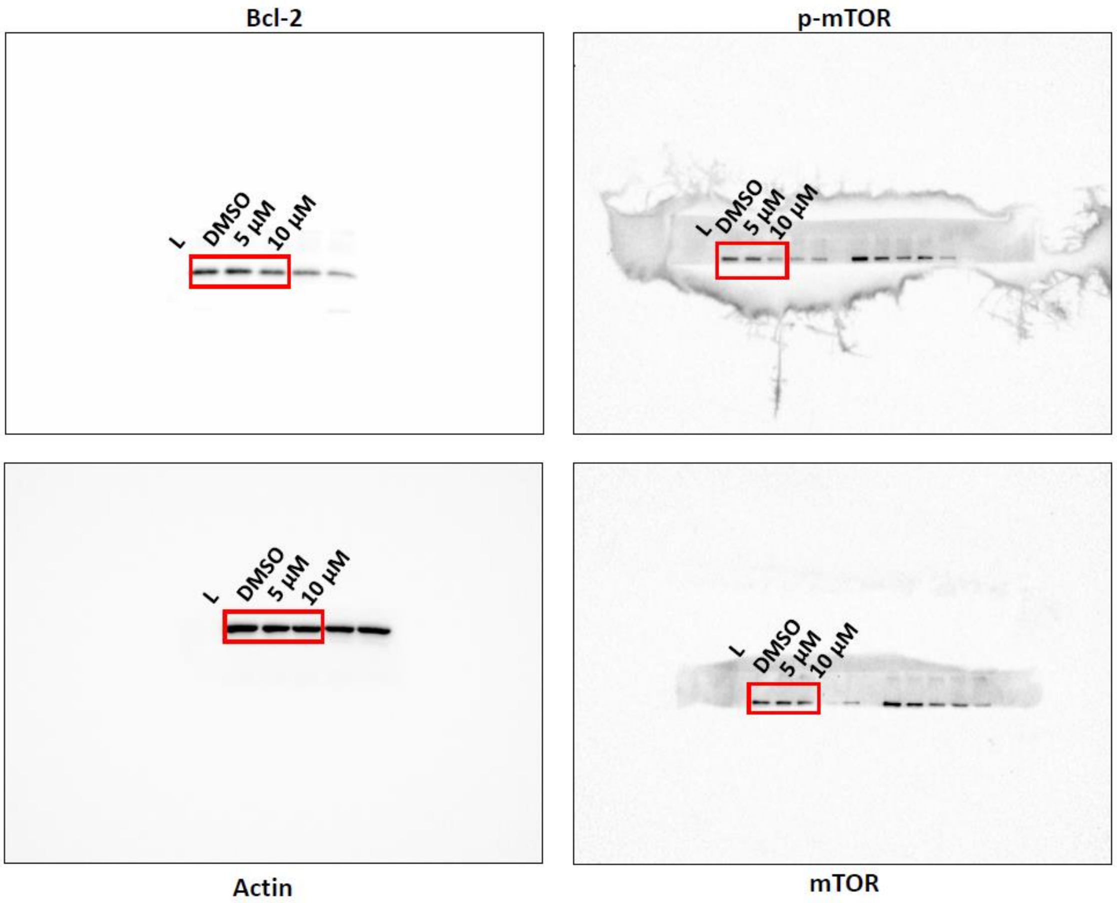

Figure S13: Whole western blot images in Figure 3A – SW480 Downstream Targets (DIM-3,5-Cl2 Treatment). Drug treatment for SW480. Red boxes indicate the cropped sections displayed in the main figure. mTOR and pmTOR were imaged before blot was cut vertically to separate two different (not same experiment) sample groups. Full blot was cut prior to primary antibody incubation to detect multiple proteins in the same blot.

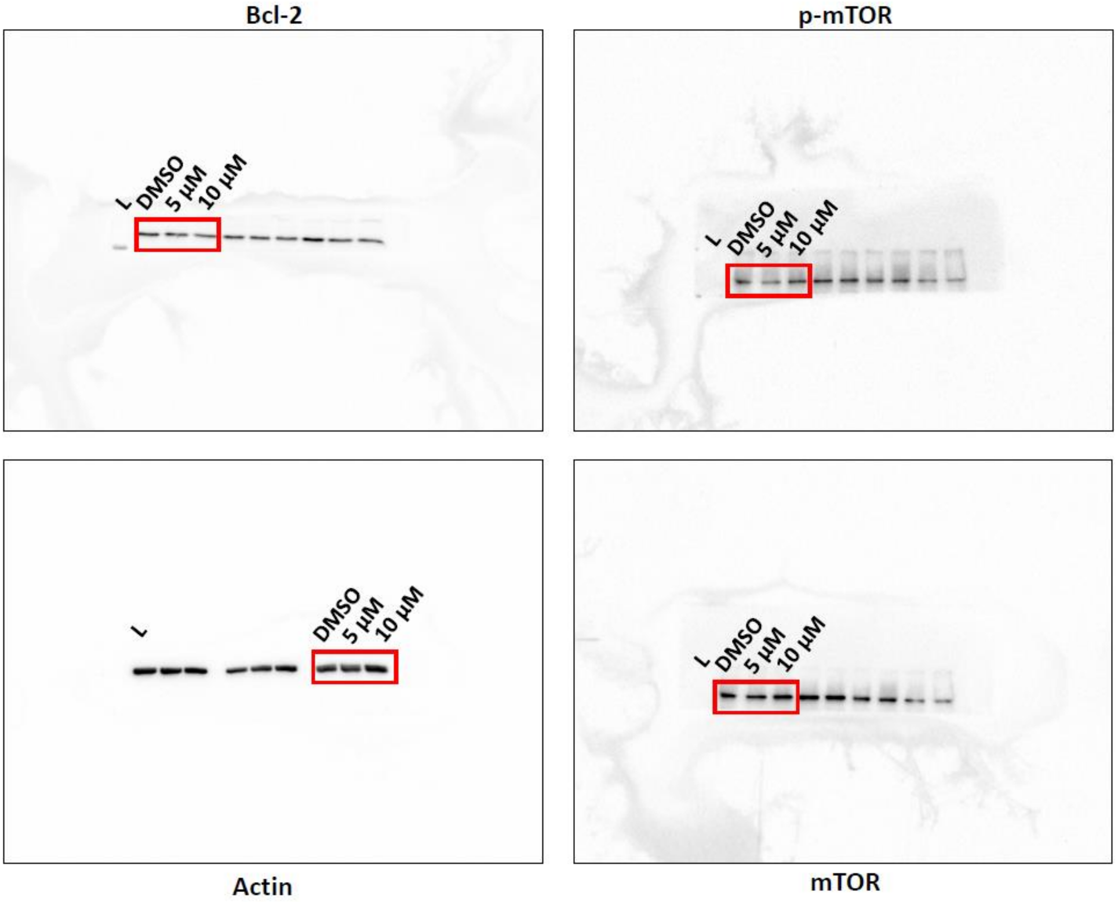

Figure S14: Whole western blot images in Figure 3A – HCT116 Downstream Targets (DIM-3,5-Cl2 Treatment). Drug treatment for HCT116. Red boxes indicate the cropped sections displayed in the main figure. Actin was generated from the same samples on a separate blot using the same loading amounts. Bcl-2, mTOR and p-mTOR were measured on the same blot.Full blot was cut prior to primary antibody incubation to detect multiple proteins in the same blot. Since mTOR and p-mTOR are of the same size, the blot was used to image mTOR first, then stripped and re-incubated for p-mTOR.

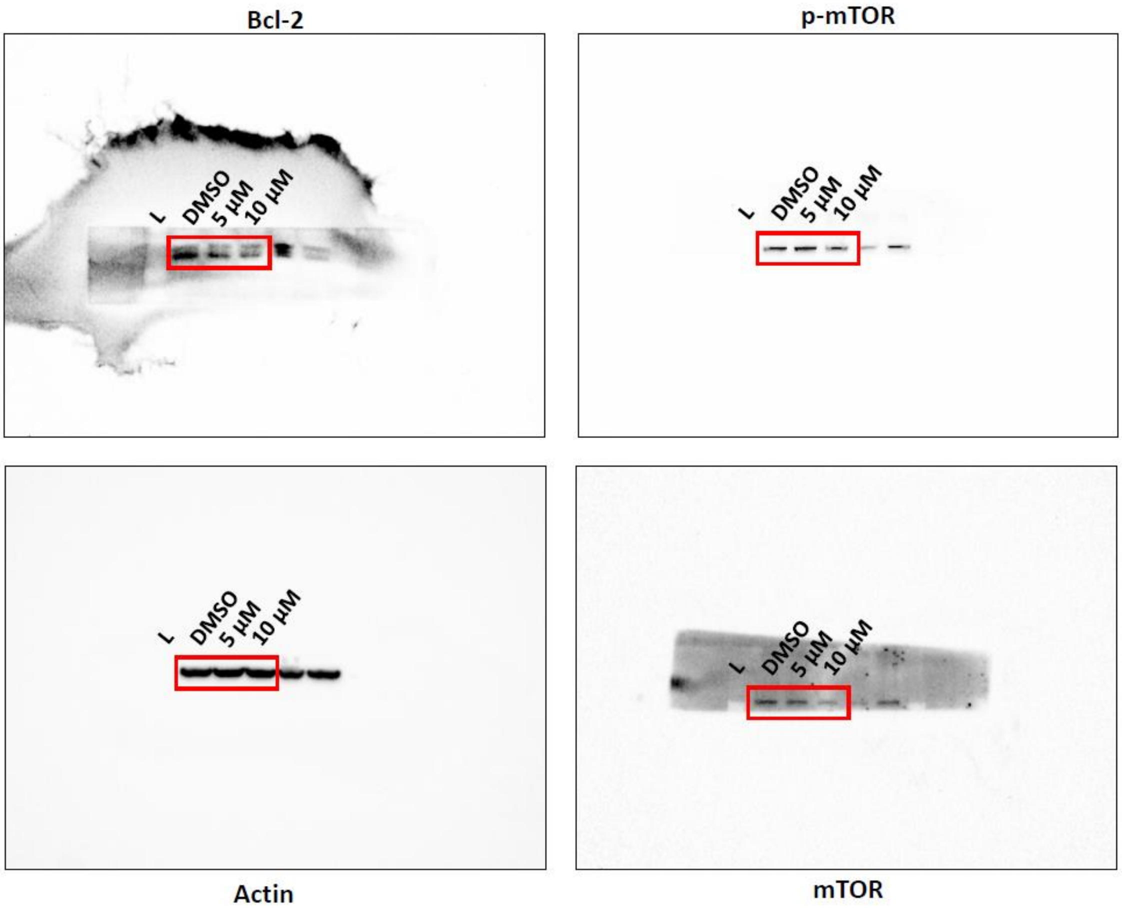

Figure S15: Whole western blot images in Figure 3A – CT26 Downstream Targets (DIM-3,5-Cl2 Treatment). Drug treatment for CT26. Red boxes indicate the cropped sections displayed in the main figure. Full blot was cut prior to primary antibody incubation to detect multiple proteins in the same blot. Since mTOR and p-mTOR are of the same size, the blot was used to image mTOR first, then stripped and re-incubated for p-mTOR.

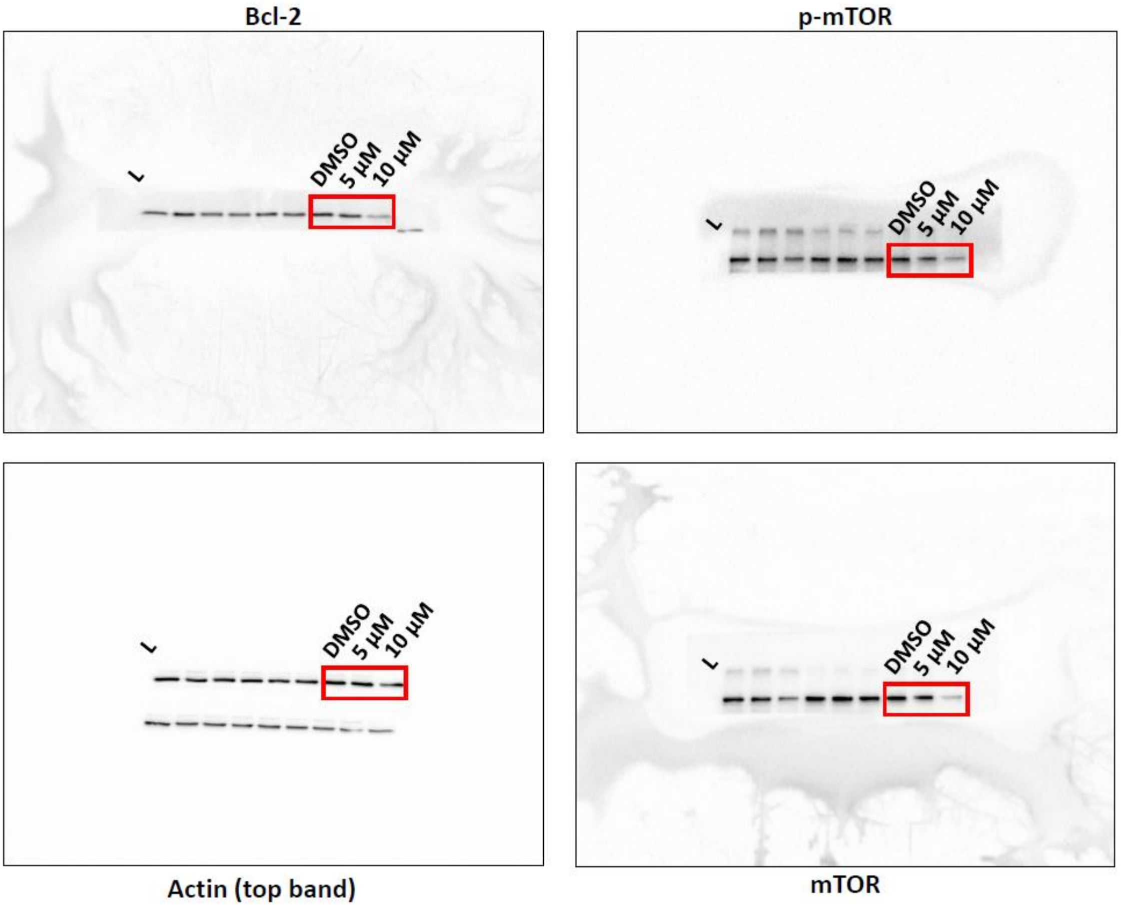

Figure S16: Whole western blot images in Figure 3E – SW480 Downstream Targets (DIM-3-Cl-5-CF3 Treatment). Drug treatment for SW480. Red boxes indicate the cropped sections displayed in the main figure. Full blot was cut prior to primary antibody incubation to detect multiple proteins in the same blot. Since mTOR and p-mTOR are of the same size, the blot was used to image mTOR first, then stripped and re-incubated for p-mTOR.

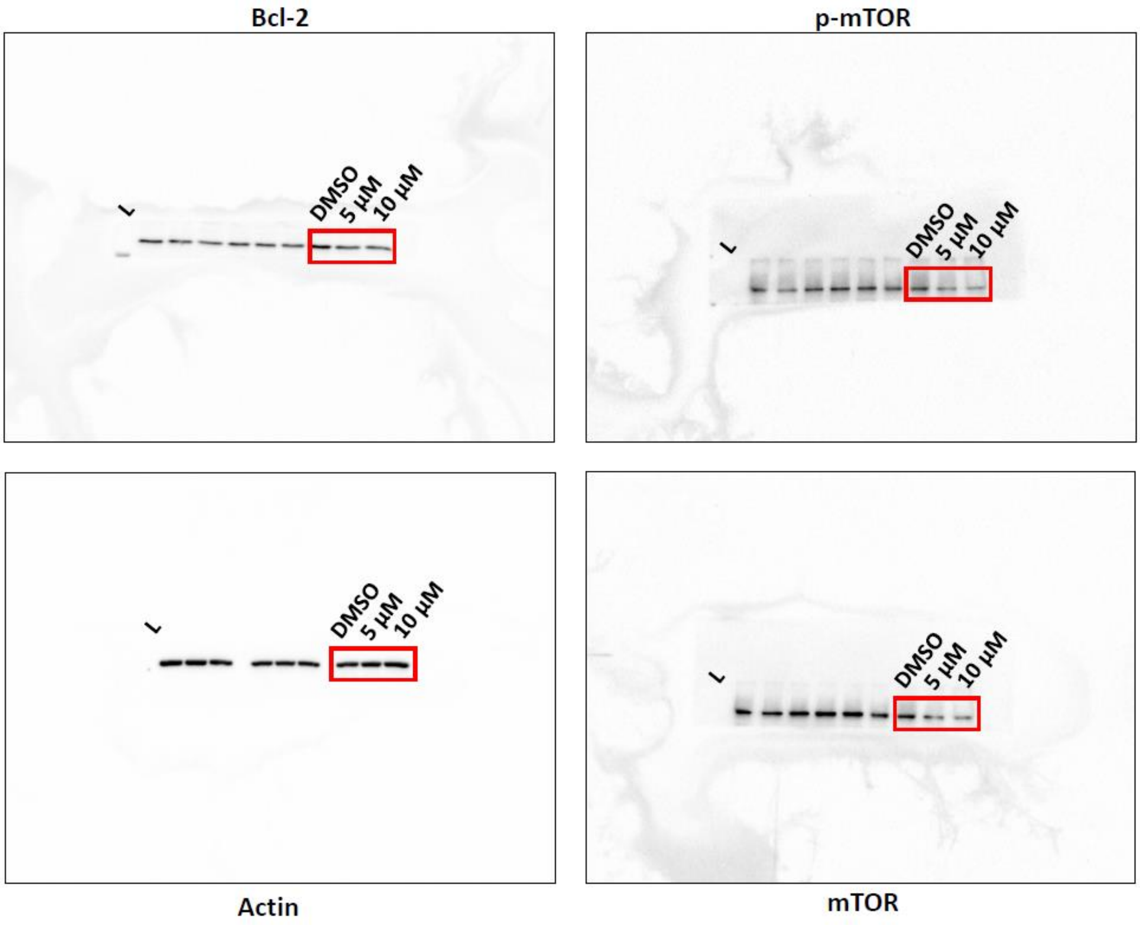

Figure S17: Whole western blot images in Figure 3E – HCT116 Downstream Targets (DIM-3-Cl-5-CF3 Treatment). Drug treatment for HCT116. Red boxes indicate the cropped sections displayed in the main figure. Actin was generated from the same samples on a separate blot using the same loading amounts. Bcl-2, mTOR and p-mTOR were measured on the same blot. Full blot was cut prior to primary antibody incubation to detect multiple proteins in the same blot. Since mTOR and p-mTOR are of the same size, the blot was used to image mTOR first, then stripped and re-incubated for p-mTOR.

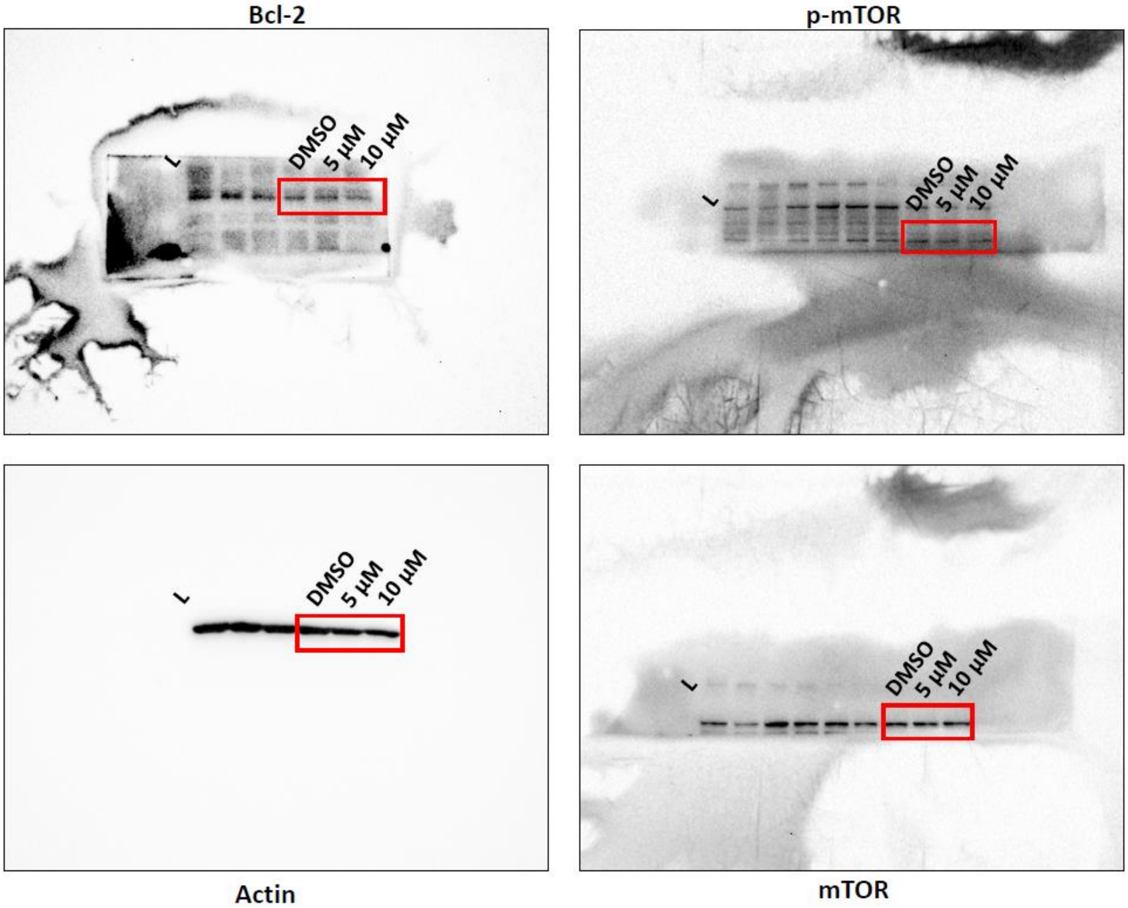

Figure S18: Whole western blot images in Figure 3E – CT26 Downstream Targets (DIM-3-Cl-5-CF3 Treatment). Drug treatment for HCT116. Red boxes indicate the cropped sections displayed in the main figure. Actin and Bcl-2 were imaged on the same blot. Same sample was used to image mTOR and p-mTOR. Full blot was cut prior to primary antibody incubation to detect multiple proteins in the same blot. Since mTOR and p-mTOR are of the same size, the blot was used to image mTOR first, then stripped and re-incubated for p-mTOR.

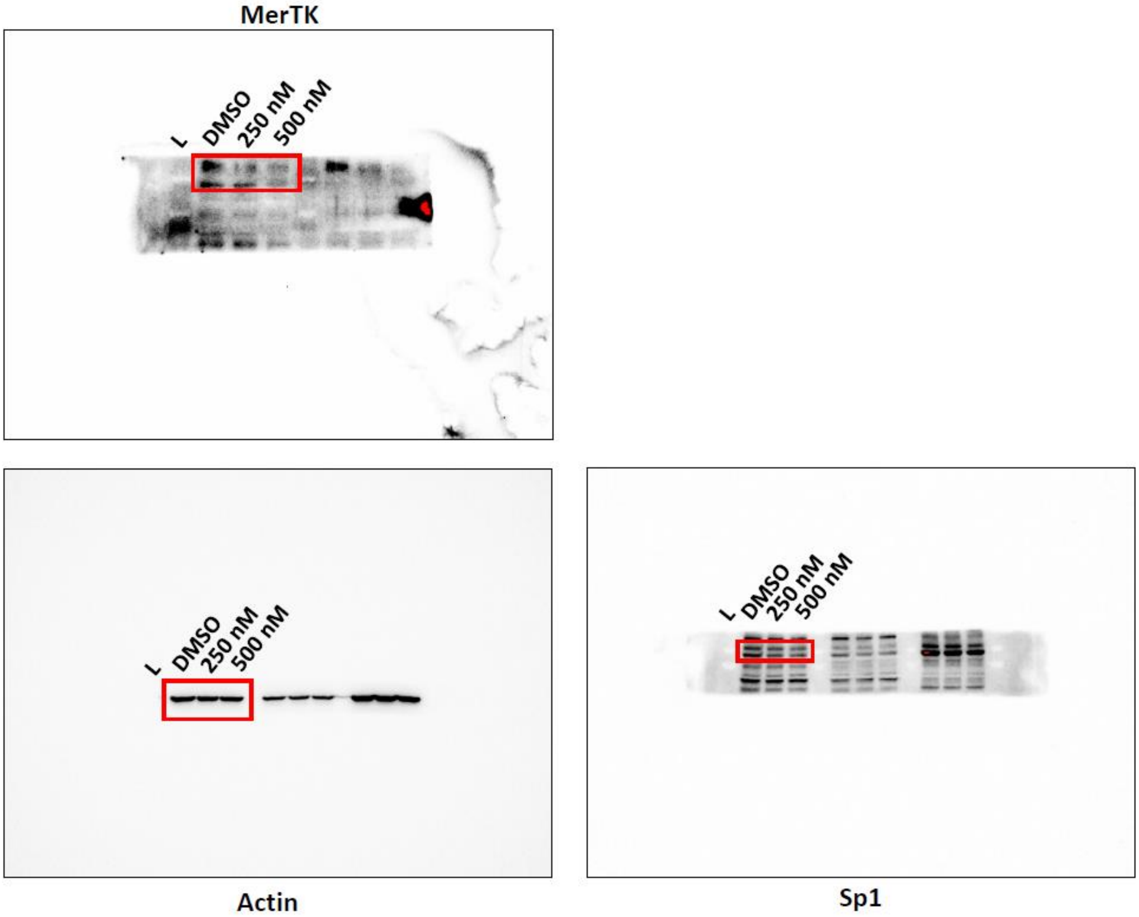

Figure S19: Whole western blot images in Figure 4B – SW480 Mithramycin Treatment. Mithramycin treatment was performed for all three colon cancer cell lines. MerTK blot was vertically cut to get more accurate imaging.

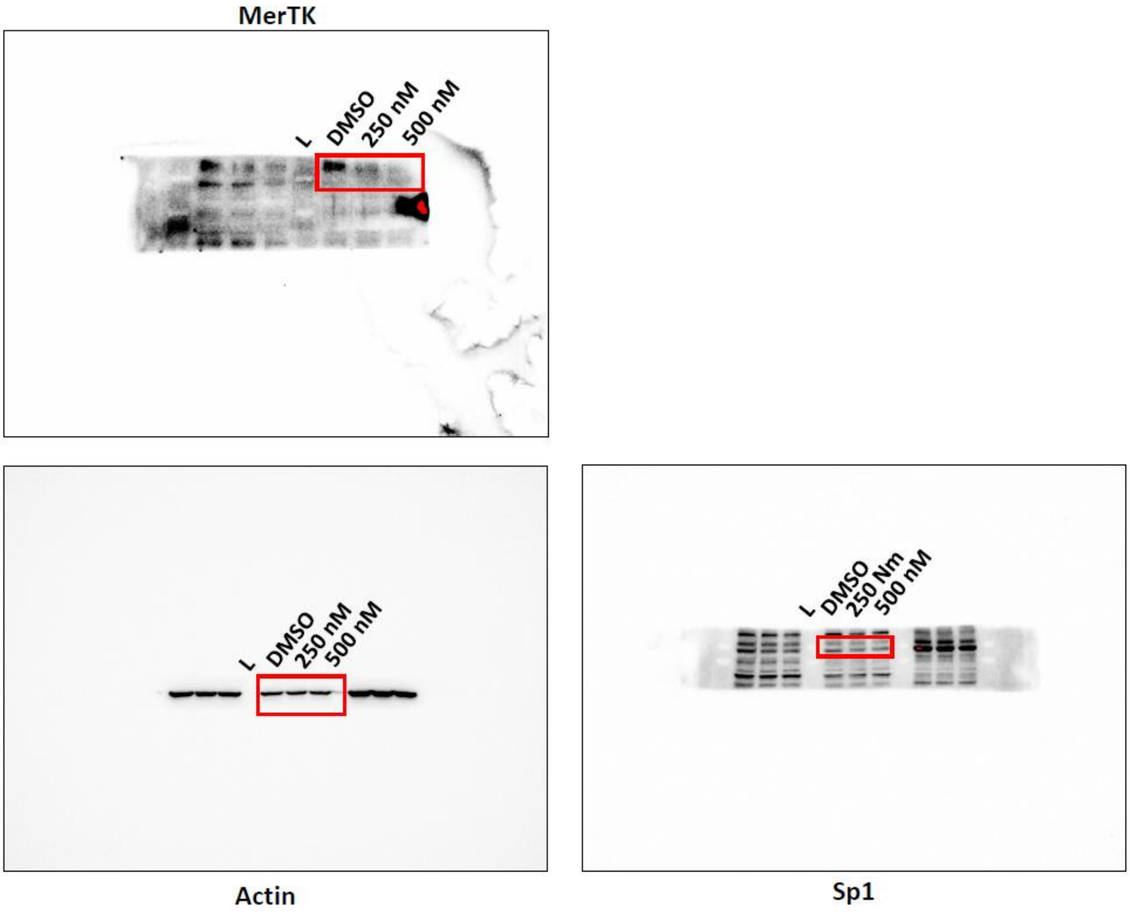

Figure S20: Whole western blot images in Figure 4B – HCT116 Mithramycin Treatment. Mithramycin treatment was performed for all three colon cancer cell lines. MerTK blot was vertically cut to get more accurate imaging.

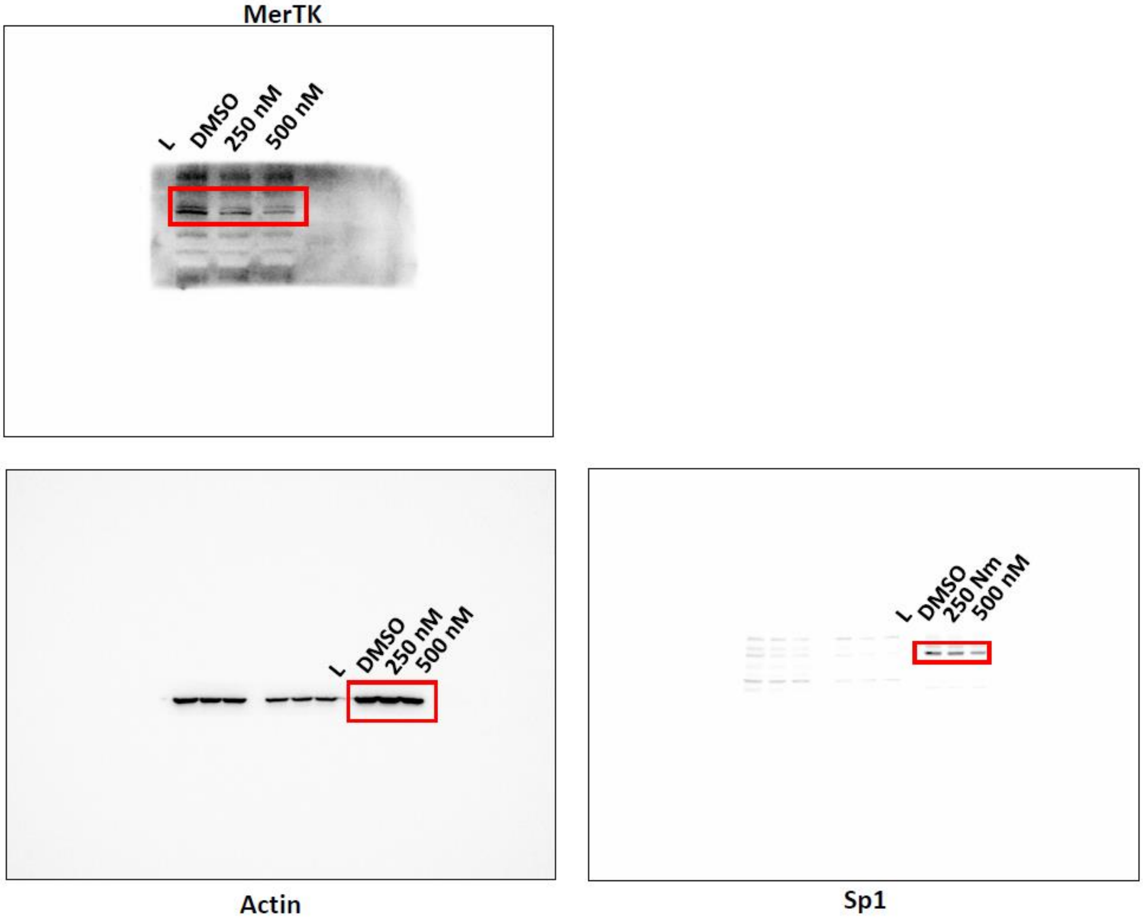

Figure S21: Whole western blot images in Figure 4B – CT26 Mithramycin Treatment. Mithramycin treatment was performed for all three colon cancer cell lines. MerTK blot is the other side of the blot on the previous side. Sp1 blot is the same as on the previous slides but re-imaging to remove over saturation.

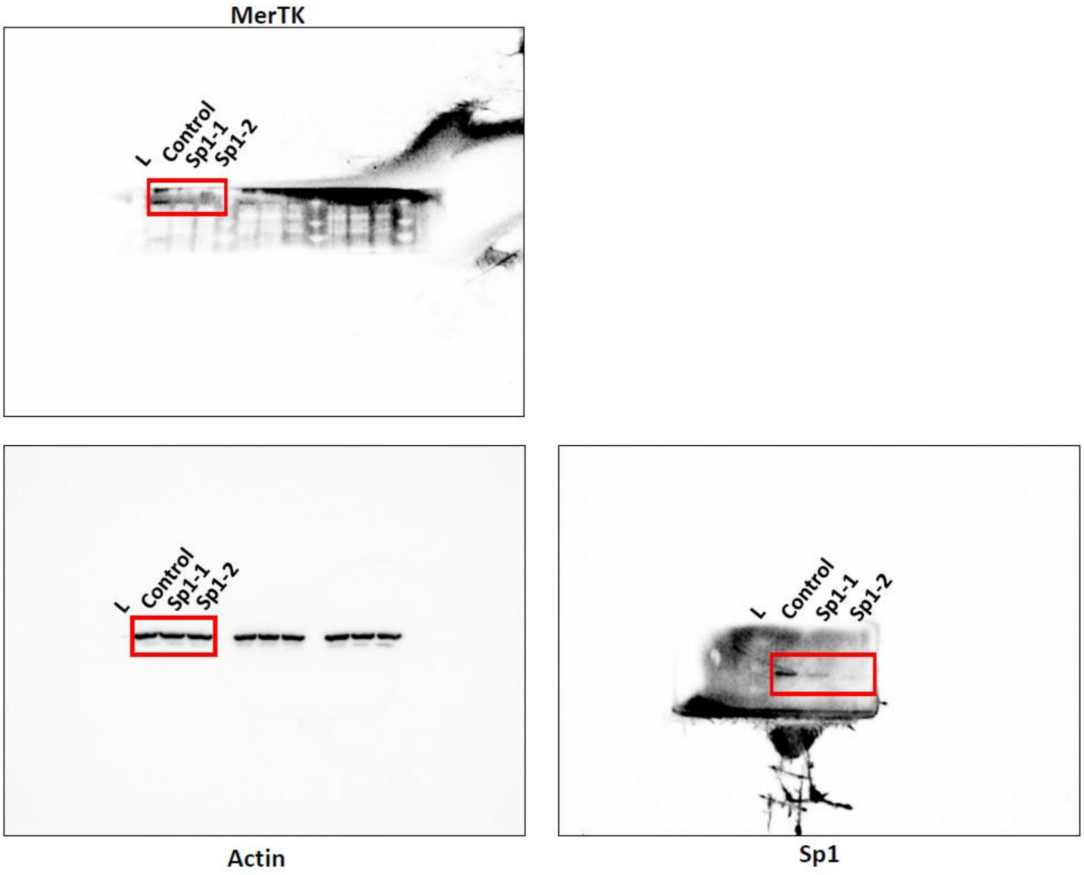

Figure S22: Whole western blot images in Figure 4D – SW480 Sp1 Knockdown Treatment. Actin, Sp1 and MerTK were run on the same blot along with other samples. The Sp region (80-100 kDa) was horizontally cut first, and then vertically cut to image for Sp1. Other regions required other antibodies.

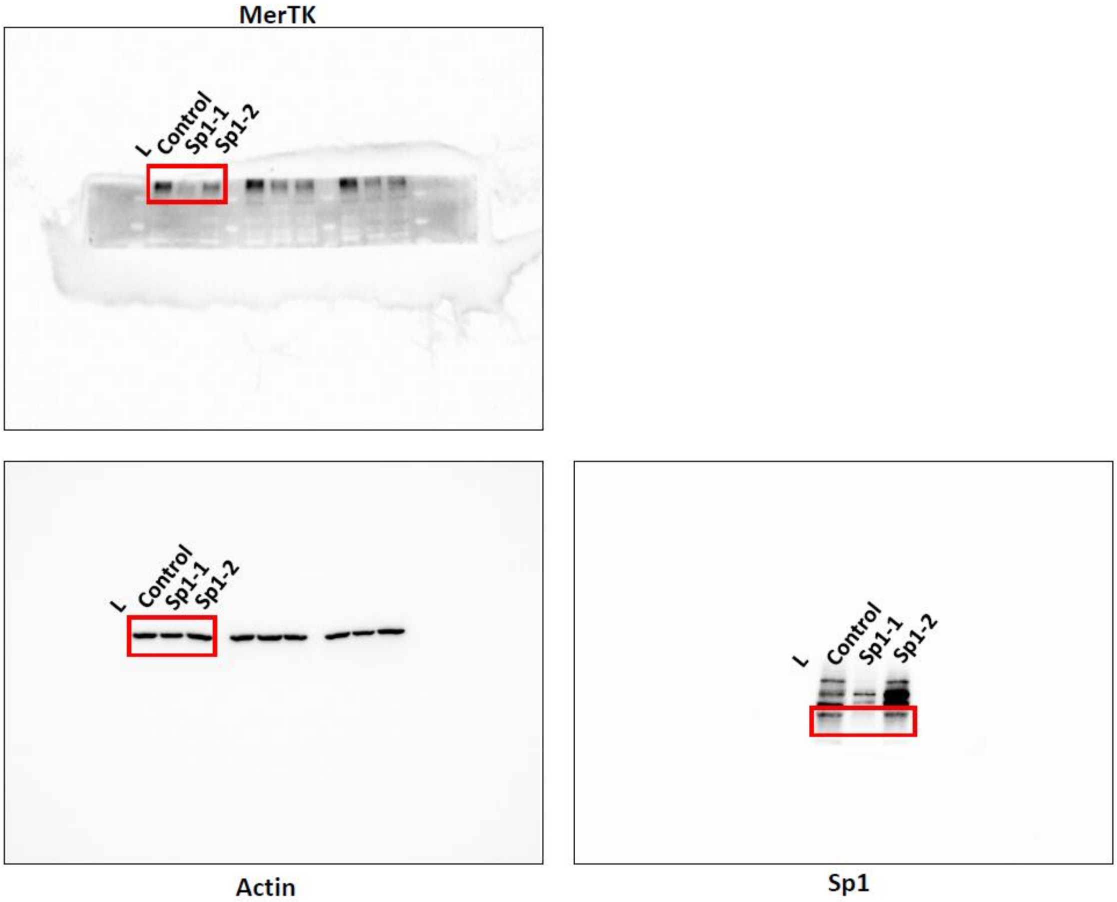

Figure S23: Whole western blot images in Figure 4D – HCT116 Sp1 Knockdown Treatment. Sp1 and Sp4 samples were run on the same blot. The region of the Sp (80-100 kDa) was first horizontally cut and isolated, and then the cut blot was further vertically cut to incubate for Sp1 separately.

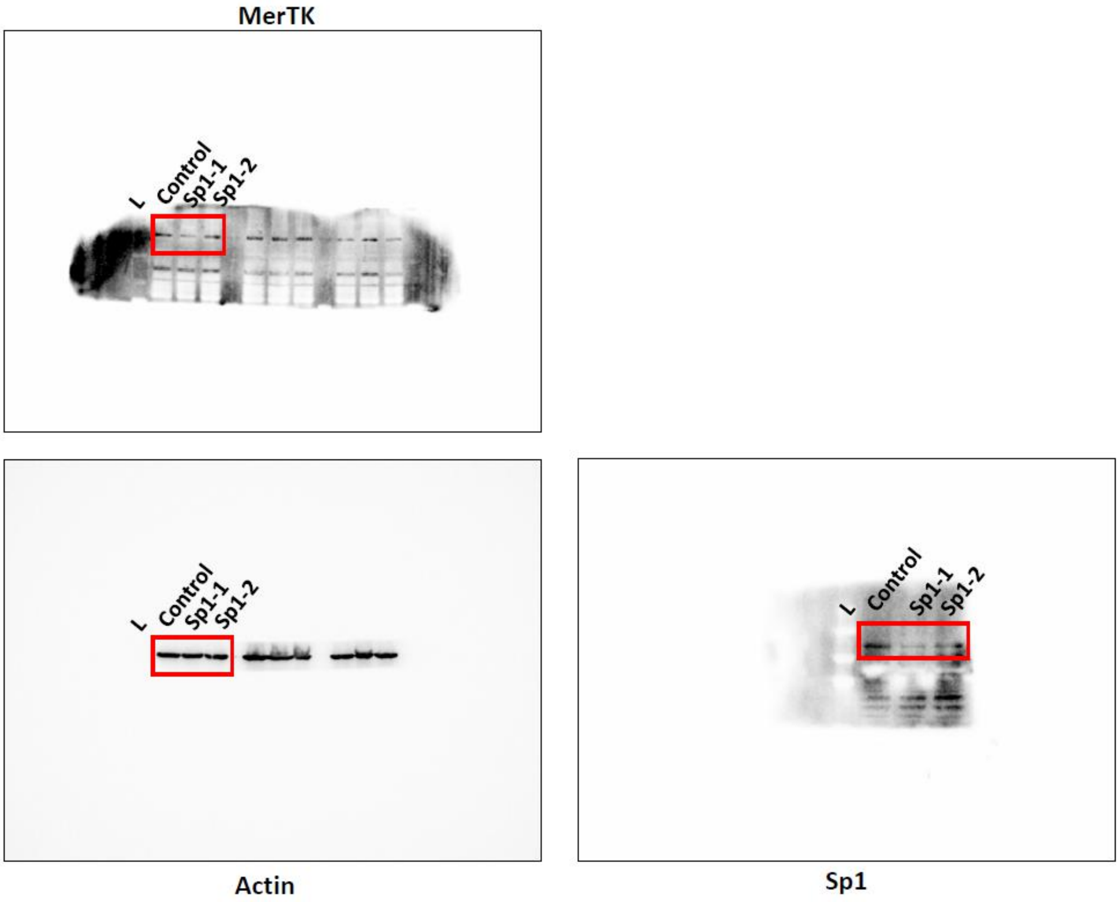

Figure S24: Whole western blot images in Figure 4D – CT26 Sp1 Knockdown Treatment. Actin, Sp1 and MerTK were run on the same blot along with other samples. The Sp region (80-100 kDa) was horizontally cut first, and then vertically cut to image for Sp1. Other regions required other antibodies.

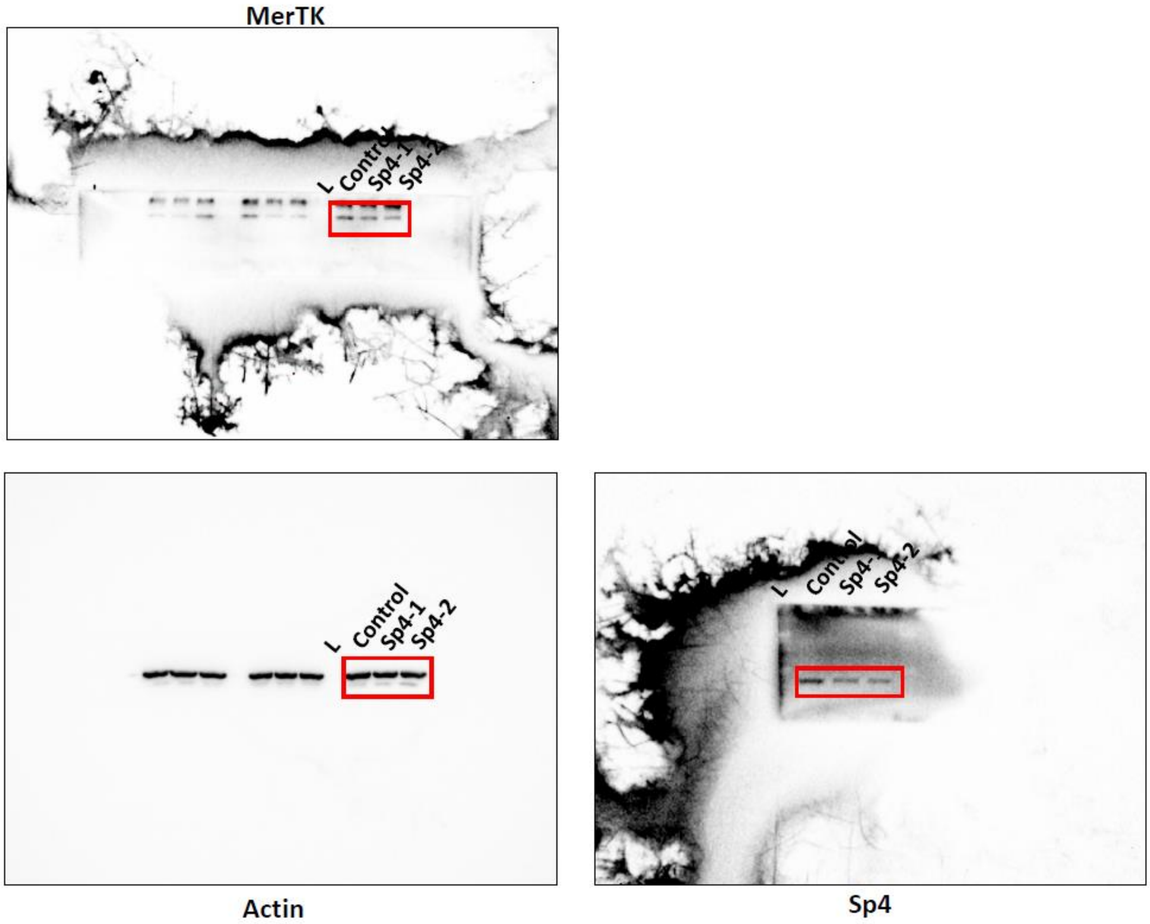

Figure S25: Whole western blot images in Figure 4F – SW480 Sp4 Knockdown Treatment. Sp1 and Sp4 samples were run on the same blot. (as seen in the Sp1 SW480 slide). The region of the Sp (80-100 kDa) was first horizontally cut and isolated, and then the cut blot was further vertically cut to incubate for Sp4 separately.

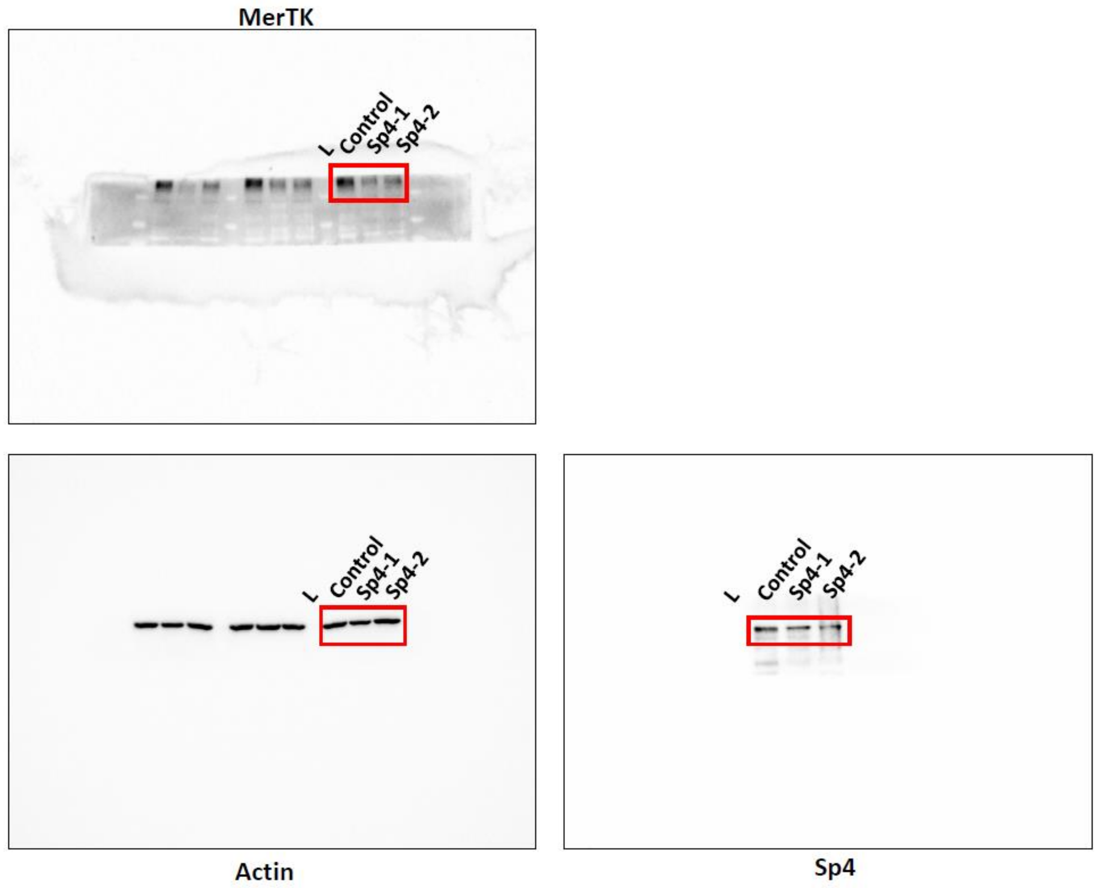

Figure S26: Whole western blot images in Figure 4F – HCT116 Sp4 Knockdown Treatment. Sp1 and Sp4 samples were run on the same blot. (as seen in the Sp1 HCT116 slide). The region of the Sp (80-100 kDa) was first horizontally cut and isolated, and then the cut blot was further vertically cut to incubate for Sp4 separately.

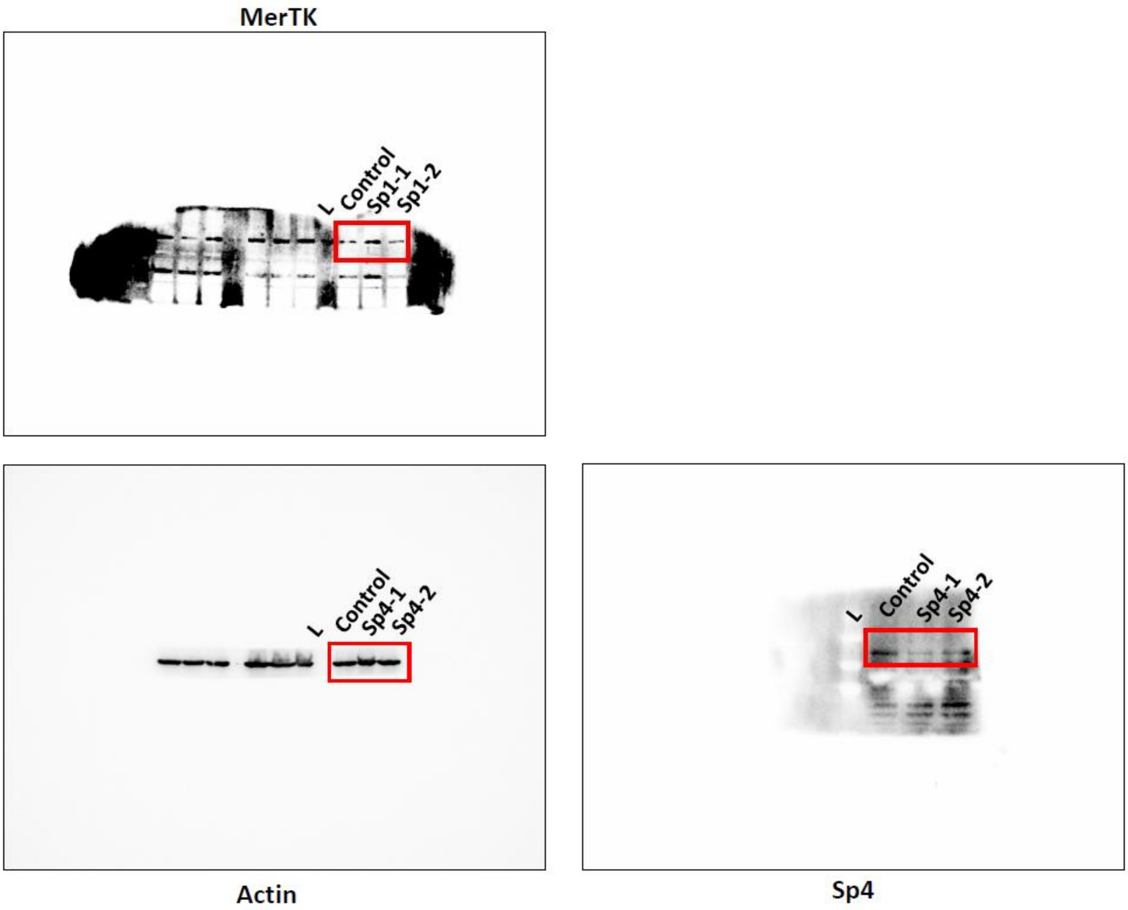

Figure S27: Whole western blot images in Figure 4F – CT26 Sp4 Knockdown Treatment. Actin, Sp4 and MerTK were run on the same blot along with other samples. The Sp region (80-100 kDa) was horizontally cut first, and then vertically cut to image for Sp4. Other regions required other antibodies.
